# Genome-wide DNA Methylation analysis in response to salinity in the model plant caliph medic (*Medicago truncatula*)

**DOI:** 10.1186/s12864-018-4484-5

**Published:** 2018-01-24

**Authors:** Mahmoud W. Yaish, Abbas Al-Lawati, Ibtisam Al-Harrasi, Himanshu Vishwas Patankar

**Affiliations:** Department of Biology, College of Science, Sultan Qaboos University, Muscat, Oman

**Keywords:** Salinity, Genomics, *Medicago truncatula*, DNA methylation, Whole-genome bisulfite sequencing, Roots, Model plant

## Abstract

**Background:**

DNA methylation has a potential role in controlling gene expression and may, therefore, contribute to salinity adaptation in plants. Caliph medic (*Medicago truncatula*) is a model legume of moderate salinity tolerance capacity; however, a base-resolution DNA methylome map is not yet available for this plant.

**Results:**

In this report, a differential whole-genome bisulfite sequencing (WGBS) was carried out using DNA samples extracted from root tissues exposed to either control or saline conditions. Around 50 million differentially methylated sites (DMSs) were recognized, 7% of which were significantly (*p* < 0.05, *FDR* < 0.05) altered in response to salinity. This analysis showed that 77.0% of the contexts of DMSs were mCHH, while only 9.1% and 13.9% were mCHG and mCG, respectively. The average change in methylation level was increased in all sequence contexts, ranging from 3.8 to 10.2% due to salinity stress. However, collectively, the level of the DNA methylation in the gene body slightly decreased in response to salinity treatment. The global increase in DNA methylation due to salinity was confirmed by mass spectrometry analysis. Gene expression analysis using qPCR did not reveal a constant relationship between the level of mCG methylation and the transcription abundance of some genes of potential importance in salinity tolerance, such as the potassium channel KAT3, the vacuolar H^+^-pyrophosphatase (V-PPase), and the AP2/ERF and bZIP transcription factors, implying the involvement of other epigenetic gene expression controllers. Computational functional prediction of the annotated genes that embrace DMSs revealed the presence of enzymes with potential cellular functions in biological processes associated with salinity tolerance mechanisms.

**Conclusions:**

The information obtained from this study illustrates the effect of salinity on DNA methylation and shows how plants can remodel the landscape of 5-methylcytosine nucleotide (5-mC) in the DNA across gene structures, in response to salinity. This remodeling varies between gene regions and between 5-mC sequence contexts. The mCG has a vague impact on the expression levels of a few selected potentially important genes in salt tolerant mechanisms.

**Electronic supplementary material:**

The online version of this article (10.1186/s12864-018-4484-5) contains supplementary material, which is available to authorized users.

## Background

Plants use several strategies to cope with high soil salinity that all require a significant modulation in gene expression through different epigenetic processes [1–3]. One of these processes is DNA methylation, in which cytosines are covalently modified by adding a methyl group to their backbone, forming a 5-methylcytosine nucleotide (5-mC) [4, 5]. Besides being a key epigenetic mark in gene silencing and playing a protective role against invading viruses [6], DNA methylation is involved in regulating normal growth and developmental processes, such as cell differentiation [7], genomic imprinting [8], X-chromosome inactivation [9], repression of repetitive elements [10] and cell senescence [11]. Additionally, in cooperation with other players, DNA methylations plays a role in epigenetic transgenerational memory which may lead to environmental adaptation [12].

Unlike animals, for which DNA methylation mainly occurs in an mCG context, plant methylomes also encompass non-mCG methylation in mCHG and mCHH contexts (where H represents any nucleotide other than G) [13]. Plants possess at least three mechanisms of DNA methylation that differ based on sequence contexts [14]. The mCG sites are maintained by DNA METHYLTRANSFERASE1 (MET1) and CHROMOMETHYLASE1 (CMT1), while the mCHG sites are maintained by CHROMOMETHYLASE3 (CMT3) however, CHH sites are maintained by constant de novo methylation by DOMAINS REARRANGED METHYLTRANSFERASE2 (DRM2) and other players in the RNA-directed DNA methylation (RdDM) pathway [14, 15].

The relationship between DNA methylation and gene expression is more complicated than was initially predicted. The effect of DNA methylation on gene expression varies based on the tissue type and the methylated sequence context, as well as on the methylated genome region within the intergenic region and the gene body [16]. In general, DNA methylation of the promoter regions often leads to reduced gene expression [17]; however, modest levels of DNA methylation within the gene body of some plants, such as Arabidopsis [18], rice [19] and maize [20], showed a positive effect on gene expression [17, 21–25].

DNA methylation can be stably inherited throughout subsequent generations, but its effect can also be eliminated or neutralized through the hydroxylation of the methyl group, at least in animals [26], however, this mechanism could not yet be found in plants because of the absence of the Ten Eleven Translocation (TET) family of enzymes that are responsible for the oxidation of 5-mC into 5-hmC [27]. Moreover plants can regulate the methylation level through demethylases of the DEMETER family such as those found in Arabidopsis [28]; and *M. truncatula* [29].

Caliph medic (*M. truncatula*) is a model plant used in basic research to study different physiological processes in legumes [30] and can endure a moderate level of soil salinity [31]. The determination of specific methylomic markers linked to a salinity tolerance trait could help to create a saline-tolerant plant through conventional plant breeding programs, as well as through direct epigenetic molecular engineering tools [21]. Based on methylation-sensitive amplified polymorphism (MSAP) and enzyme-linked immunosorbent assay (ELISA) analyses [32], the mCG methylation levels in *M. truncatula* were substantially increased due to salinity stress [31]. However, the global methylomic landscape, including the identity of the affected genes, the degree of methylation and the redistribution of each sequence context in the genome in response to salinity stress, were yet to be identified. The aim of this study was to construct a methylome map for *M. truncatula* and determine changes occur in the methylation status in response to salinity. Therefore, in this project, single-base resolution genome-wide mapping of cytosine methylation and an in silico functional analysis of the methylated genes of *M. truncatula* methylome were carried out using bisulfite combined with Illumina sequencing and computational technologies.

## Methods

### Plant materials and growth conditions

The plants were grown in pots and treated with NaCl solution, as previously described [31]. Briefly, seeds of *Medicago truncatula* were surface-sterilized and planted in 5-L pots and kept in a greenhouse under natural light/dark conditions, and 30 and 25 °C during the day and night, respectively. When reached the pre-flowering mature growth stage (9 weeks post-sowing), the plants were irrigated for 1 week either with distilled water (control) or with 20 dS/m (204 mM) NaCl solution. DNA samples were separately extracted from a pool of roots of ten saline-treated and untreated (control) *M. truncatula* plants using the QIAGEN genomic DNA extraction kit, following the manufacturer’s protocol. Each pool of the DNA sample was considered as a single biological replicate which was used for WGBS following the previously implemented strategy in other plant species [20, 33, 34]. The quality and quantity of DNA were verified using the Qubit® method. The level of soil salinity was measured based on the saturated soil paste extract using an electrical conductivity (EC) meter.

### Methyl-maxiSeq™ library construction

WGBS techniques were carried out as previously described [34]. The DNA libraries were constructed, sequenced and bioinformatically analyzed at the Zymo Research (ZR) Laboratories, California, USA, which was the service provider. Methyl-MaxiSeq™ libraries were prepared from 500 ng of genomic DNA digested with 2 units of ZR’s dsDNA Shearase™ Plus (Cat. number E2018–50). The fragments produced were end-blunted and 3′-terminal-A extended, then purified using the ZR DNA Clean & Concentrator™-5 kit (Cat. number D4003). The A-tailed fragments were ligated to pre-annealed adapters containing 5′-methyl-cytosine, rather than cytosine, and the adapter-ligated fragments were filled in. Bisulfite treatment of the fragments was performed using the EZ DNA Methylation-Lightning Kit (ZR, Cat. number D5030). PCR was carried out with Illumina TruSeq indices and the size and concentration of the fragments were confirmed on the Agilent 2200 TapeStation, then pair-end sequenced with 50 read length of each end using Illumina HiSeq 1500 platform. Each sequence pair was separately aligned and then the alignment files were merged for each read. This alignment method was chosen because for Methyl-maxiSeq™ samples, single-end alignment gives slightly better results than paired-end.

### Methyl-maxiSeq™ sequence alignments and data analysis

The sequence reads from bisulfite-treated EpiQuest libraries were identified using standard Illumina base-calling software and then analyzed using a ZR proprietary analysis pipeline, written in Python and using Bismark v 0.14.3 [35] as the alignment software for analysis. Index files were constructed by a bismark_genome_preparation command using the entire *M. truncatula* as a reference genome (medTrv.4.0v2) and available at (http://jcvi.org/medicago) website. After removal of the duplicate reads, unique best alignments were retained and the methylation ratios from the alignment file across the gene and the promoter regions were calculated. The Bismark v0.14.3 with bowtie 1 (bt1) software was used to align and calculate the algorithm for the methylation ratios from the alignment file across the gene and the promoter regions. In this analysis, the methylation ratio was defined as the measured number of 5-mCs divided by the total number of cytosines covered at the particular site. In general, sites were categorized as hypermethylated if a site within the genome of the salinity-treated plant was significantly (*p* < 0.05, *FDR* < 0.05) more methylated than the control, and were categorized as hypomethylated if a site within the genome of the salinity-treated plant was significantly less methylated than the control.

The promoter and gene regions were defined as those respectively plus and minus 3 Kb from the transcription start site (TSS). This region included the transcription end sites (TES). The gene database and gene feature boundaries were obtained from the Gramene resources [36] available at (http://www.gramene.org/). Non-directionality and other default parameters were applied while running Bismark. The methylation level of each sampled cytosine was estimated as the number of reads reporting a C, divided by the total number of reads reporting a C or T. The level of DNA methylation across the genes was profiled. The methylation ratios were plotted against gene structural features, including the promoter, gene body and flanking regions, which include TSS, TES, and 3 Kb up- and down-stream regions. Fisher’s exact test was performed for each cytosine with a minimum coverage of five aligned sequence reads based on *p* < 0.05. Only significant (*p* < 0.05, *FDR* < 0.05) differentially methylated sites (DMSs) were considered for subsequent analysis. Hypo/hypermethylation status was assigned to each DMS based on the difference in the methylation ratio between the two samples. If the methylation ratio of a DMS of the control sample was higher than the methylation ratio of the same DMS of the NaCl-treated sample then the DMS was considered as a hypomethylated site in response to salinity treatment. However, if the methylation ratio of a DMS of the control sample was lower than the methylation ratio of the same DMS of the NaCl-treated sample then the DMS was considered as a hypermethylated site in response to salinity treatment. The promoter, gene body and mCG island annotations were added when available. The methylation percentage at each particular site was viewed using the Integrative Genomics Viewer (IGV) browsers.

The change in methylation level of each DMS for each sequence contexts was calculated separately based on the following formula: The methylation ratio of the control sample (C) was subtracted from the methylation ratio of the NaCl-treated sample (T) and the resulted number was divided by the methylation ratio of the control sample (C), “(T–C)/C”. Subsequently, the average change in methylation levels of all DMSs were collectively calculated for each sequence contexts.

### Gene ontologies and annotations

Strongly methylated genes were used in this analysis. The top 2000 sites with the highest methylation changes including strongly hyper- or hypomethylated at the mCG, mCHG and mCHH sites were functionally annotated based on the similarity of the protein-coding mRNA sequence to other proteins available in the GenBank databases. The protein sequences were classified based on biological processes, cellular components and molecular functions using the Blast2GO PRO software package [37]. Differential functional enrichment analysis between the hyper- and hypomethylation for a particular site (mCG, mCHG or mCHH) were identified using Fisher’s exact test and based on *p* ≤ 0.001. Mapping of the coding protein sequences within the metabolic pathways was carried out using the Kyoto Encyclopedia of Genes and Genomes (KEGG) [38] tools, implemented within the Blast2GO PRO software.

### Identification of differentially methylated regions (DMRs)

Pairwise comparison of the methylation profiles for the control and treated samples was carried out for different annotated gene regions. The DMRs were calculated by subtracting the methylation ratio of a region within the genome of the control sample from the methylation ratio of the same region within the genome of the salinity treated sample. If the calculated value was positive, then the DMR was considered as hypermethylated. However, if the calculated value was negative, then the DMR was considered as hypomethylated. In this analysis, a 50-bp sliding window was used to identify the DMRs between the control and the salinity-treated samples. The comparison was done for the three methylated sequence contexts (mCG, mCHG or mCHH). Significantly methylated regions (*p* < 0.001, *FDR* ≤ 0.05) were selected for further analysis. The circos software [39] was used to construct the chromosomes Circos plots.

### Global DNA methylation and hydroxymethylation analysis using mass spectrometry

An aliquot of 400 ng of each genomic DNA sample extracted from the root tissues of the saline-treated and control plants was digested to single nucleotides using DNA Degradase Plus (Zymo Research, Irvine, CA). A selected reaction monitoring (SRM)-based mass spectrometry assay was used to quantify 5-hydroxymethyl-2′-deoxycytidine (5HmdC) and 5-methyl-2′-deoxycytidine (5mdC). The assay was designed to measure concentrations of 5HmdC and 5mdC as a percentage of 2′-deoxyguanosine (dG). The calibrated ranges for the components were 0–7.5% for 5HmdC and 0–75% for 5mdC using a fixed 40 pmol amount of dG as an internal standard, as previously described [40]. Both calibration curves had an r^2^ value greater than 0.9997. Replicates for the unknown samples were run in triplicate and followed by a blank to eliminate carryover into the next unknown run. Samples were gradient eluted from a 2.1 × 100 mm Hypersil GOLD aQ C18 reversed-phase column with a guard cartridge, using an Accela 600 quaternary pump at 250 ul/min and a column temperature of 40 °C. The analysis was conducted on a Thermo Scientific TSQ Vantage Triple Quadrupole mass spectrometer; previously determined transitions at 258.2 → 141.93 m/z for 5HmdC, 242.2 → 126.10 m/z for 5mdC and 268.1 → 152.3 m/z for dG were monitored, with dwell times of 0.375 s each and scan widths of 0.002 m/z. The collisional gas pressure was set at 1.2 mTorr. The raw data were processed and evaluated within the Xcalibur Qual Browser using ICIS peak detection. Baseline windows of 30 units were used for 5mdC and 5HmdC, while windows of 40 units were used for dG. The peak detection was set for the highest peak option and the minimum signal-to-noise ratio for all analytes was 3.0.

### cDNA synthesis and quantitative real time PCR (qPCR)

The expression levels of selected genes of potential importance in salinity tolerance mechanisms were investigated using the qPCR. Total RNA was extracted from the three biological replicates of root tissues of NaCl-treated and control plants using the QIAGEN Plant RNA Extraction Kit. Each biological replicate was composed of a pool of 10 plants. Genomic and organelle DNAs were eliminated from the total RNA using the on-column DNA digestion method and the QIAGEN RNase-free DNase according to manufacturer’s instructions. In addition to the Nanodrop spectrophotometry, the quality and the quantity of the RNA were checked using 1% TAE agarose gel electrophoresis. The 5 μL RNA was converted to cDNA using the SuperScript™ IV First-Strand Synthesis System kit (Invitrogen, USA) in a 20 μL reaction, according to the manufacturer’s instructions. Specific primers were designed using the Primer Express 3.0.1 software (Applied Biosystem, USA), using default parameters: optimum primer length 20 bases, minimum length nine bases and maximum length 40 bases; maximum melting temperature 60 °C and minimum 58 °C; maximum amplicon length 150 bp and minimum 50 bp; maximum GC content 80% and minimum 30%. The qPCR reaction was carried out using the 7500 Fast Real-Time PCR System (Applied Biosystem, USA) in a total volume of 10 μL per reaction containing 5 μL Fast SYBR® Green Master Mix (Applied Biosystem, USA), 0.1 μL of each primer (100 pmol/μL) (Additional file 1: Table S1), 2 μL diluted cDNA and 2.8 μL nuclease-free water. The reaction conditions used for the qPCR reaction were as follows: 95 °C for 20 s followed by 40 cycles of 95 °C for 3 s and 60 °C for 30 s. The qPCR was carried out using the MIQE guidelines [41]. The 2^−ΔΔCT^ [42] method was used to calculate the gene expression, while actin 11 (GenBank accession number TC85697) and O-linked N-acetylglucosamine transferase (also called secret agent) (GenBank accession number TC77416) genes were used as the reference genes for normalization of gene expression as previously used [43, 44]. To determine the statistical significance of the gene expression ratios, the qPCR data was analyzed using the online statistical analysis tool BootstRatio [45].

## Results

### WGBS and the next-generation sequencing platform

Global bisulfite conversion was employed together with the next-generation sequencing method to detect the single-based resolution and relative amount of 5-mCs changes across the genome of *M. truncatula* when grown under control and saline conditions. The analysis resulted in a total of 637,685,284 read pairs and 17,426,891 unique mCGs obtained from sequencing DNA samples extracted from root tissues of the control and salinity-treated plants. The reads sequenced from the control and salinity-treated plants were mapped onto the genome with an efficiency of 26% and 21% for the DNA extracted from the control and salinity-treated plants, respectively (Table 1).

**Table 1:** Bisulfite sequencing results in read pairs (RP) from the DNA extracted from the roots of *M. truncatula,* grown under control or saline conditions

	Total RP	Unique mCGs	Unique mCHGs	Unique mCHHs	Mapping efficiency
Control	318,711,344	8,937,395	10,917,437	73,555,016	26%
NaCl-Treated	318,973,940	8,489,496	10,320,643	69,069,219	21%
Total	637,685,284	17,426,891	21,238,080	142,624,235	

### Differential methylation status of the mCG, mCHG and mCHH sites

**Fig. 1:**
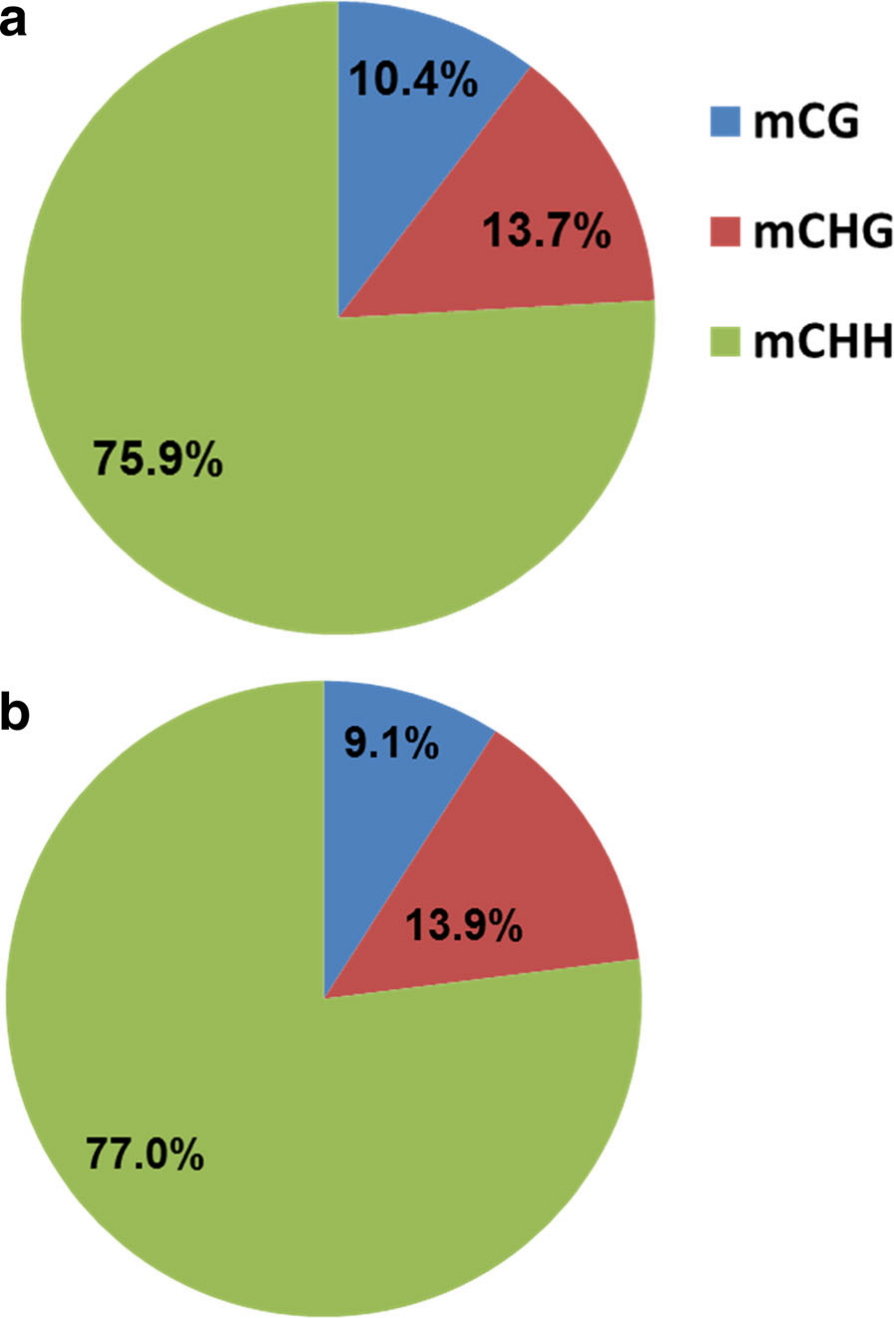
The percentage of the total methylated sequence of each context (**a**) and the percentage of the significantly (*p* < 0.05) methylated sequence of each context obtained in this study (**b**)

Genome-wide analysis of the 5-mCs was screened and scored based on the methylation ratios. The sequence analysis revealed the presence of 49,630,860 methylated sites, where 14.9% of these were significantly (*p* < 0.05, *FDR* < 0.05) altered in response to salinity stress. The analysis also showed that three-quarters of the identified context sequences were mCHH, while the rest were either mCHG or mCHH (Fig. 1a). The statistical analysis showed that the majority (77.0%) of the significantly (*p* < 0.05, *FDR* < 0.05) methylated sites were mCHH, while only 9.1% of sites were mCG and 13.9% were mCHG (Fig. 1b). Regardless of the methylated sequence context, the DMSs showed an increase in methylation levels due to salinity treatment. This increase ranged between 3.8 to 10.2% in the methylation level of the significantly (*p* < 0.05, *FDR* < 0.05) DMSs which were covered with at least five reads, due to salinity (Table 2).

**Table 2:** A summary of the methylome data obtained from genome-wide profiling. Total number of methylated sites in control and salinity-treated samples including those of insignificantly abundance changes (^a^), and significantly abundance changes (^b^) based on *p* < 0.05, *FDR* < 0.05

Methylation context	Total number of 5-mC^a^	Hypermethylated 5-mCs in control sample^b^	Hypermethylated 5-mCs in NaCl-treated sample^b^	Total number of DMSs^b^	Average change in methylation level (%)^b^
mCG	5,751,299	497,714	298,540	796,254	3.8
mCHG	6,590,349	562,527	483,057	1,045,584	8.9
mCHH	37,289,212	3,103,541	2,693,344	5,796,885	10.2
Total	49,630,860	4,163,782	3,474,941	7,638,723	

To obtain information about the identity of the strongly methylated/demethylated genes to a significant level (*p* < 0.05, *FDR* < 0.05) due to salinity treatment, the uppermost hyper- and hypomethylated DMSs (top 100 or 2000) within the genome of the control and the NaCl-treated plants of each specific context (mCG, mCHG, mCHH) were selected for further analysis.

### DNA methylation profile of functional genes, promoters and chromosomes

**Fig. 2:**
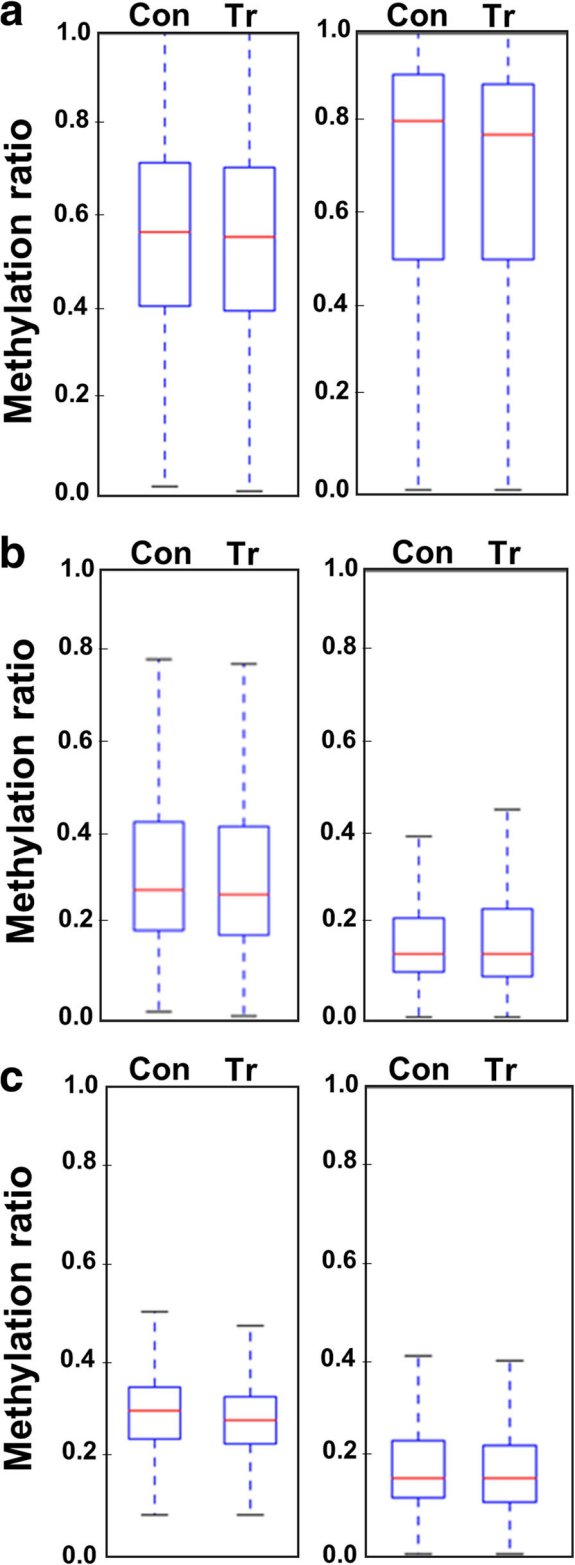
Box plots display the overall methylation level of significantly (*p* < 0.05, *FDR* ≤ 0.05) methylated sites measured as methylation ratios among promoter (left panel) and coding regions (right panel), under control (Con) and salinity stress conditions (Tr). mCGs are shown in **a**, mCHGs in **b**, and mCHHs in **c**. A line within the bars represents the median

An overview of the DNA methylation levels using box plots showed that the methylation ratio at the mCG was the highest among the other methylated sequence contexts (Fig. 2). A comparative look at that level revealed that the mCG methylation ratio was higher at the coding regions than the promoters (Fig. 2a). However, the methylation levels of the mCHG at the coding regions were lower than those found in the promoters (Fig. 2b). A similar situation was also found when mCHH was profiled (Fig. 2c). It was observed that the methylation ratios at the mCG of the promoters and the coding regions were slightly reduced in response to salinity (Fig. 2a). This minor reduction in response to salinity was also observed in the mCHG and the mCHH of the promoter region (Fig. 2b and c).

**Fig. 3:**
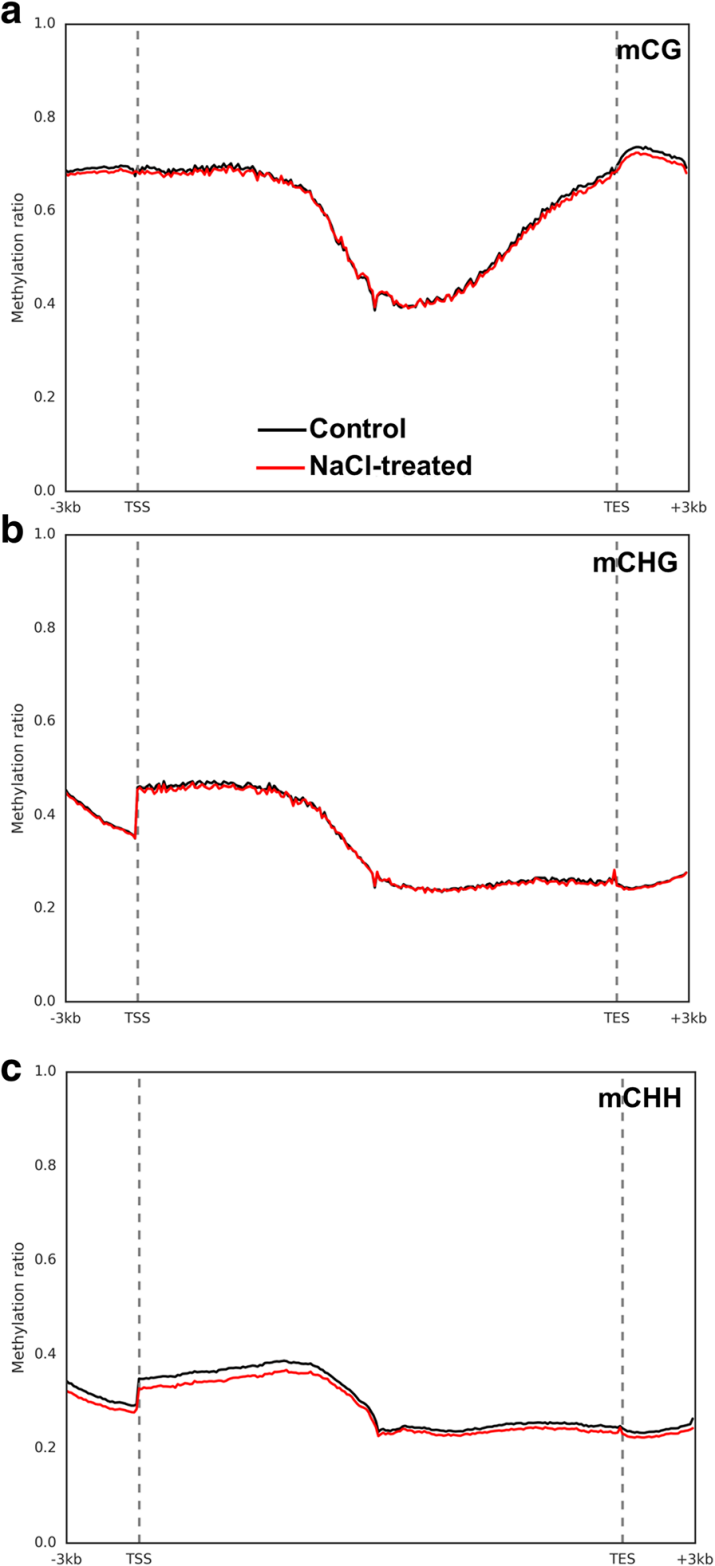
The level of DNA methylation profiles across the gene features including the promoter, gene body and flanking regions, which include transcription start site (TSS), transcription end site (TES), and 3 Kb up- and down-stream regions. DNA methylation at the mCG, mCHG, and mCHH sites are shown in panels **a**, **b**, and **c**, respectively

The level of the 5-mCs within the putative functional genes and their promoters was collectively assessed in the genome extracted from NaCl-treated and control root tissues. The DNA methylation profile showed that the NaCl treatment slightly decreased the mCG and mCHG methylation levels at the promoters and transcript regions, yet this decline was more obvious at the mCHH sites (Fig. 3). The results also showed that the DNA methylation level of the mCG was the highest among the sequence contexts, followed by mCHG and then mCHH. The methylation profile of the promoter regions (upstream from the TSS) revealed that the DNA methylation for the mCG sequence context was relatively high, and this level was consistent with the flanked region of the transcript, yet this level decreased in the center of the transcript before incrementally increasing to the topmost level in the region downstream from the TES region (Fig. 3a). In comparison with the transcript region, the promoter regions at the mCHG and mCHH sites were relatively hypomethylated, yet the level of DNA methylation dramatically increased at the TSS region and subsequently declined in the rest of the transcript and toward the downstream regions of the TES (Fig. 3b and c).

**Fig. 4:**
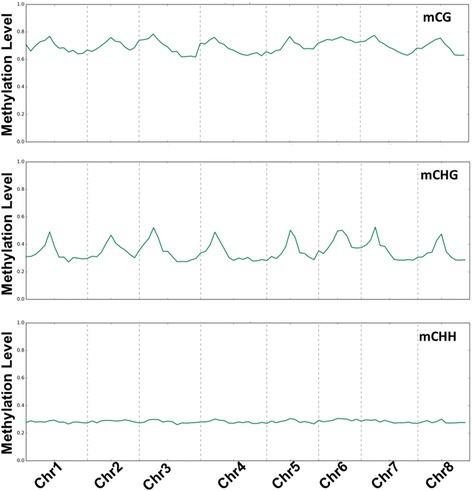
Chromosome-level view of the methylation percentage of different cytosine sequence contexts

In order to determine the distribution of the 5-mCs across the *M. truncatula* genome and the effect of the salinity treatment on the level of DNA methylation, mCG, mCHG and mCHH sequence contexts of the eight chromosomes were estimated based on the ratio of reads reported as 5-mCs. The results showed that mCG acquired the highest level of methylation, while mCHH showed the lowest methylation level among the sequence contexts as indicated by the heights of the peaks in the scatterplot. The whole chromosome plot of the mCG and mCHG sites also revealed that the level of DNA methylation reached the maximum level around the center of each chromosome however, mCHH showed an almost constant level of methylation across the chromosomes. In addition, potentially telomere region of chromosome six and seven showed relatively higher levels of mCG and mCHG methylation than the other chromosomes (Fig. 4). In this analysis, pairwise comparison of the methylation profiles did not show clear differences in the methylation levels between saline-treated and control samples at the chromosomal level.

**Fig. 5:**
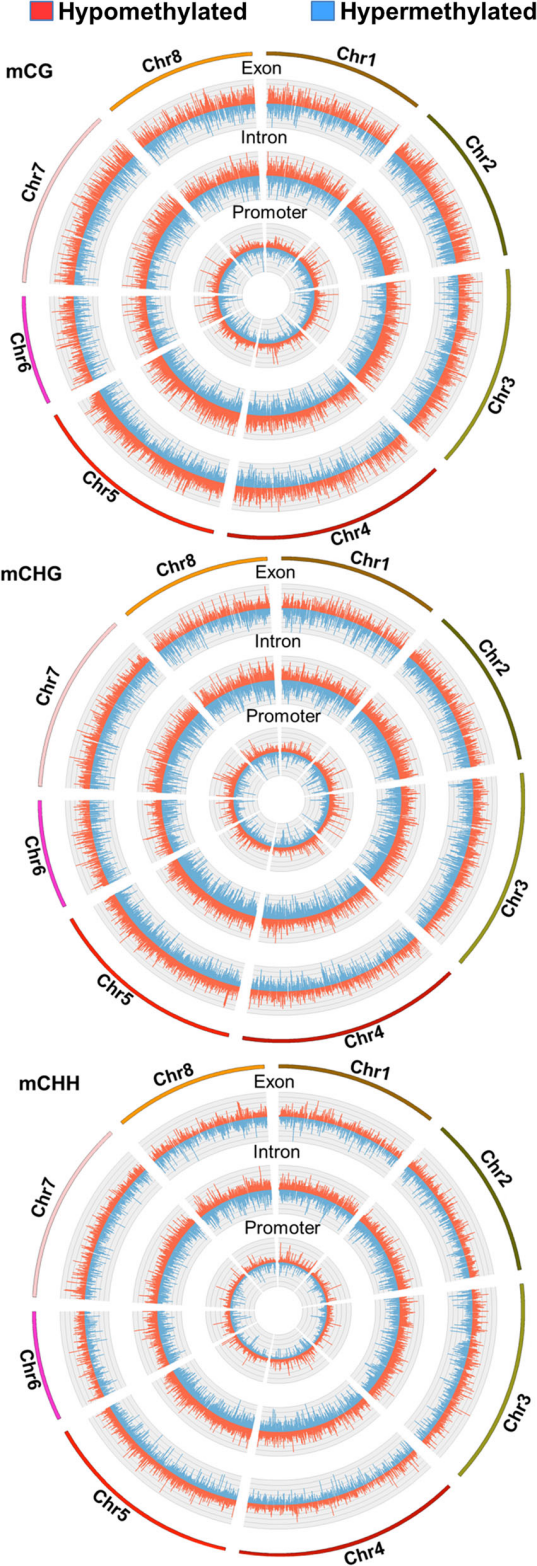
Whole-genome pairwise methylation analysis showed as circos plots. Significantly (*p* < 0.001, *FDR* ≤ 0.05) differentially methylated regions (DMRs) on the histograms were presented using 50-bp as a comparison window

Circos plot demonstration of the hypo and hypermethylated mCG, mCHG and mCHH regions within the exons, introns and promoters of the eight chromosomes showed that the mCG methylation was distributed at the highest densities and peaks heights among the other sequence contexts followed by mCHG (Fig. 5). The circos plot showed also that the number of the strongly methylated DMRs localized within promoters was lower than those regions found in the exons and the intron regions (Fig. 5). Indeed, the sequence analysis of the DMRs showed that the number of strongly methylated mCG regions was higher than the regions embraced other sequence contexts (Table 3). The analysis also showed that while the effect of salinity on the number of DMRs varies based on the sequence contexts within the gene, the number of strongly DMRs within the promoter was slightly changed in response to salinity regardless to the sequence contexts (Table 3).

**Table 3:** The number of DMRs observed after salinity treatment

Sequence contexts	All sites	Total significant^*^	Hypomethylated^*^	Strongly hypomethylated^*^	Hypermethylated^*^	Strongly hypermethylated^*^	Total strongly methylated DMRs^*^
mCG-exon	158,359	48,104	27,910	3077	15,067	2050	5127
mCHG-exon	180,623	53,794	28,921	1352	21,319	2202	3554
mCHH-exon	191,379	77,208	46,557	617	29,205	829	1446
mCG-intron	100,915	28,258	16,345	2359	8150	1404	3763
mCHG-intron	127,043	36,217	19,031	1487	13,478	2221	3708
mCHH-intron	140,256	60,857	36,648	1060	21,903	1246	2306
mCG- promoter	52,208	20,188	12,085	150	7790	163	313
mCHG- promoter	52,350	19,109	11,923	153	6911	122	275
mCHH- promoter	52,968	31,209	23,239	57	7870	43	100
Total	1,056,101	374,944	222,659	10,312	131,693	10,280	20,592

Significantly DMRs were calculated based on *p* < 0.001, *FDR*  ≤ 0.05, and indicated by the asterisks (*). DMRs were identified using 50-bp as a comparison window

Since global methylation analysis of the genes and promoter region is unlikely to reveal a particular relationship between salinity treatment and DNA methylation changes, two groups of genes embraced highly DMSs (top 100 or 2000) were further investigated.

### Cluster analysis of the top 100 methylated sites

Since highly methylated sites are potentially important in several cellular transcriptomic activities, the top 100 differentially methylated mCG, mCHG and mCHH sites identified from DNA extracted from the control and treated plants were clustered based on methylation profile similarity. The dendrograms of hierarchical clustering showed the two DNA samples had a different DNA methylation pattern at the mCG, mCHG and mCHH sites. While the majority of mCG and mCHG sites were significantly (*p* < 0.05, FDR < 0.05) hypermethylated in the genome extracted from plants grown under control conditions, the majority of the mCHH sites were hypermethylated in the genome extracted from plants exposed to salinity stress (Additional file 2: Figure S1).

### Distribution of 5-mCs among the different gene features in the top 2000 DMSs

**Fig. 6:**
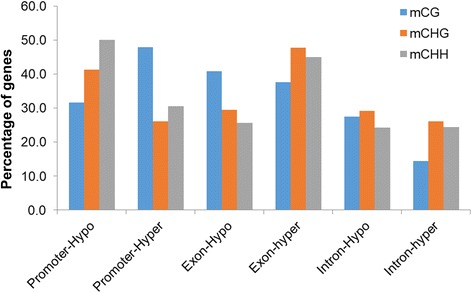
The distribution of different methylated contexts among the gene features in response to salinity stress

In this study, an increase in the methylation due to salinity was considered as a hypermethylation situation. The top 2000 DMSs of each sequence context (mCG, mCHG and mCHH) located within the different annotated gene features (promoter, exon and intron) were classified based on their methylation status. The results revealed that the mCHH context was the main methylated site within the promoters of the genes when plants were grown under normal conditions, while the mCG context was the main in the same region for those plants grown under salinity stress (Fig. 6). The methylation context mCG occupied the highest percentage among the contexts found in the exons of the DNA when plants were grown under normal conditions, while the methylated mCHG and mCHH contexts occupied the highest percentages among the contexts found in the exon regions in when exposed to salinity. Regardless of the salinity treatment, the intron regions were enriched with mCHG sites (Fig. 6).

**Fig. 7:**
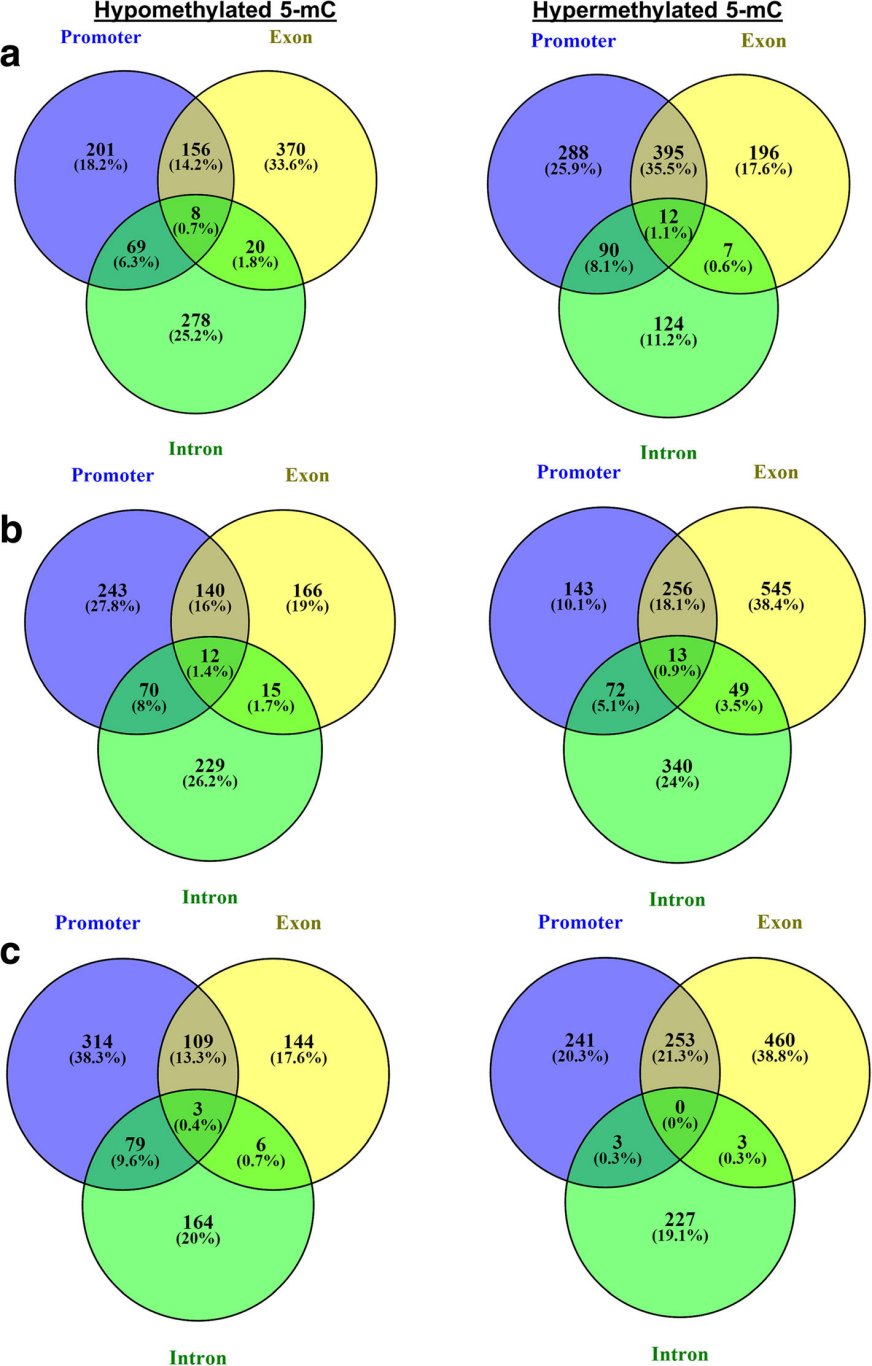
Pie distribution of the DMSs among the gene features of the top 2000 altered genes by methylation for the mCG (**a**), mCHG (**b**) and mCHH (**c**) sequence contexts

It was observed that only 1% or less of the studied genes of different predicted functions were covered by 5-mCs throughout the entire gene structures (Fig. 7). However, for the majority of the annotated genes, the methylation level and distribution varied among the gene structures and methylation contexts. For example, 18.2% of the hypomethylated mCG sites were located within the promoter, 33.6% within the exon and 25.2% within the intron region. Meanwhile, the position of 25.9% of the hypermethylated mCG was found within the promoter, 17.6% within the exon and 11.2% within the intron regions; 35.5% of the sites were hypermethylated in both the promoter and exon regions (Fig. 7a). On the other hand, 27.8% of the annotated genes were hypomethylated at the mCHG site located within the promoter, 19% within the exon and 26% within the intron region. However, 38.4% of the genes were hypermethylated in the exon region in response to salinity stress (Fig. 7b). Interestingly, the highest percentage of hypomethylated promoter regions was within the mCHH context, where it accounted for 38.3% of the studied genes (Fig. 7c). In general, at the promoter regions, a high percentage of genes were hypermethylated in the mCG context in response to salinity stress, while the mCHH and mCHG contexts were relatively hypomethylated under the same conditions (Fig. 7).

### Functional annotation of the top 2000 methylated sites

To obtain a comprehensive account of the function of those genes that were significantly altered by DNA methylation when *M. truncatula* roots were exposed to salinity stress, the function of those genes that included any of the top 2000 DMSs was predicted based on the homology of their coding DNA sequence with those proteins available in public databases. The functional annotation results showed that most of the coding genes belonged to the functions of metabolism, oxidation-reduction and regulation of the transcription process (Additional file 3: Figure S2), and were involved in integral components of the membrane, nucleus, plasma and vacuolar membranes and endoplasmic reticulum (Additional file 4: Figure S3), mainly functioning in cellular protein, ion, ATP and nucleic acid-binding activities (Additional file 5: Figure S4).

**Fig. 8:**
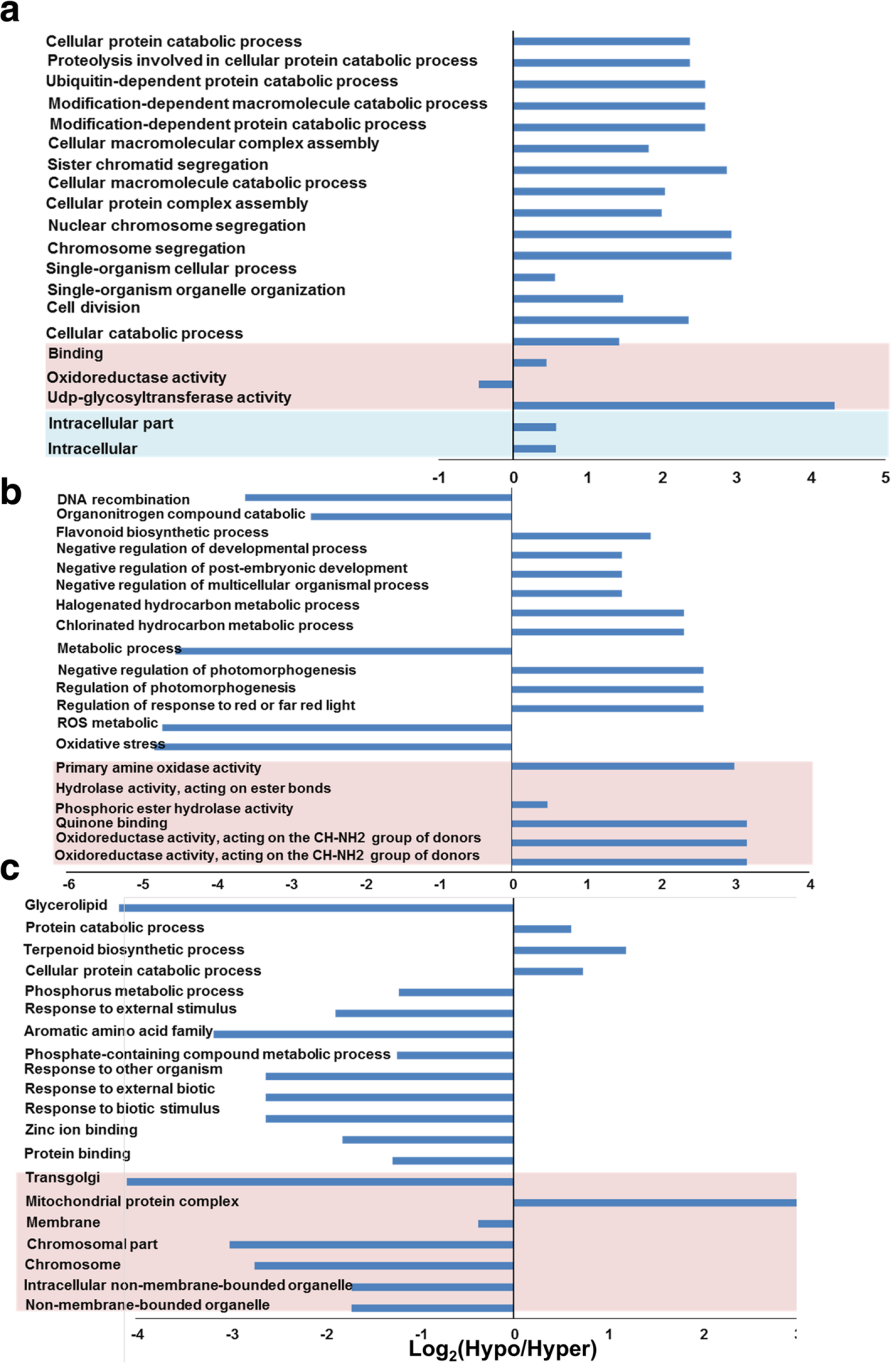
Fisher’s exact test for the significant (*p* < 0.001) enrichment analysis of gene ontology terms of the biological process, cellular components and molecular function categories of the annotated genes based on the DMSs hypermethylated sequences used as a test group in mCG (**a**), mCHG (**b**) and mCHH (**c**) DMSs

To differentiate between the abundance of ontology terms among the hyper- and hypomethylated genes when plants were grown under saline conditions, a differential enrichment analysis was carried out based on Fisher’s exact test, with *p* ≤ 0.001. This analysis was carried out separately for each of the mCG, mCHG and mCHH methylation contexts. The results showed that hypomethylated mCG was enriched in all top-scored ontology categories except oxidoreductase activity (Fig. 8a); hypomethylated mCHG was enriched in the majority of the gene ontologies except DNA recombination and response to organonitrogen compound catabolic, hydrogen peroxide metabolic and reactive oxygen species metabolic processes (Fig. 8b). However, hypermethylated mCHH was enriched in the majority of the gene ontologies except protein catabolic, terpenoid biosynthetic and cellular protein catabolic processes, and the mitochondrial protein complex cellular component (Fig. 8c).

Functional annotation analysis of the significantly methylated sequences showed that these genes encoded 1511 enzymes, including 532, 540 and 439 enzymes, mCG, mCHG and mCHH, respectively (Additional file 6: Tables S2, Additional file 7: Tables S3, Additional file 8: Tables S4). Classification of the differentially methylated genes based on enzyme categories revealed these genes belonged to the hydrolase, isomerase, ligase, lyase, oxidoreductase and transferase enzyme classes (Additional file 9: Figure S5). Glycosphingolipid biosynthesis (both lacto and neolacto) and polyketide sugar unit biosynthesis were among the pathways specifically found in genes embracing mCG (Additional file 6: Table S2); lipopolysaccharide biosynthesis, D-Alanine, D-Arginine and D-Ornithine metabolisms were among those pathways specifically found in genes embracing mCHG (Additional file 7: Table S3); and glycosaminoglycan biosynthesis (chondroitin sulfate/dermatan sulfate) and lipoic acid metabolism were among those pathways specifically found in genes embracing mCHH (Additional file 8: Table S4). The functional annotation of genes simultaneously harboring DMSs across their promoter, exon and intron regions demonstrated that these genes were coding for protein families of a function unrelated to salinity tolerance in plants. However, the functional annotation of the coding regions of the DMSs of all sequence contexts located in at least one of the gene regions revealed the presence of various deduced amino acid sequences, with a potential function in salinity tolerance, such as ion transporters and antiporters [2], abscisic acid (ABA) and proline regulators and producers [46] (Additional file 10: Table S5, Additional file 11: Table S6 and Additional file 12: Table S7).

### DNA methylation at mCG sites on the promoter region did not consistently affect gene expression levels

**Fig. 9:**
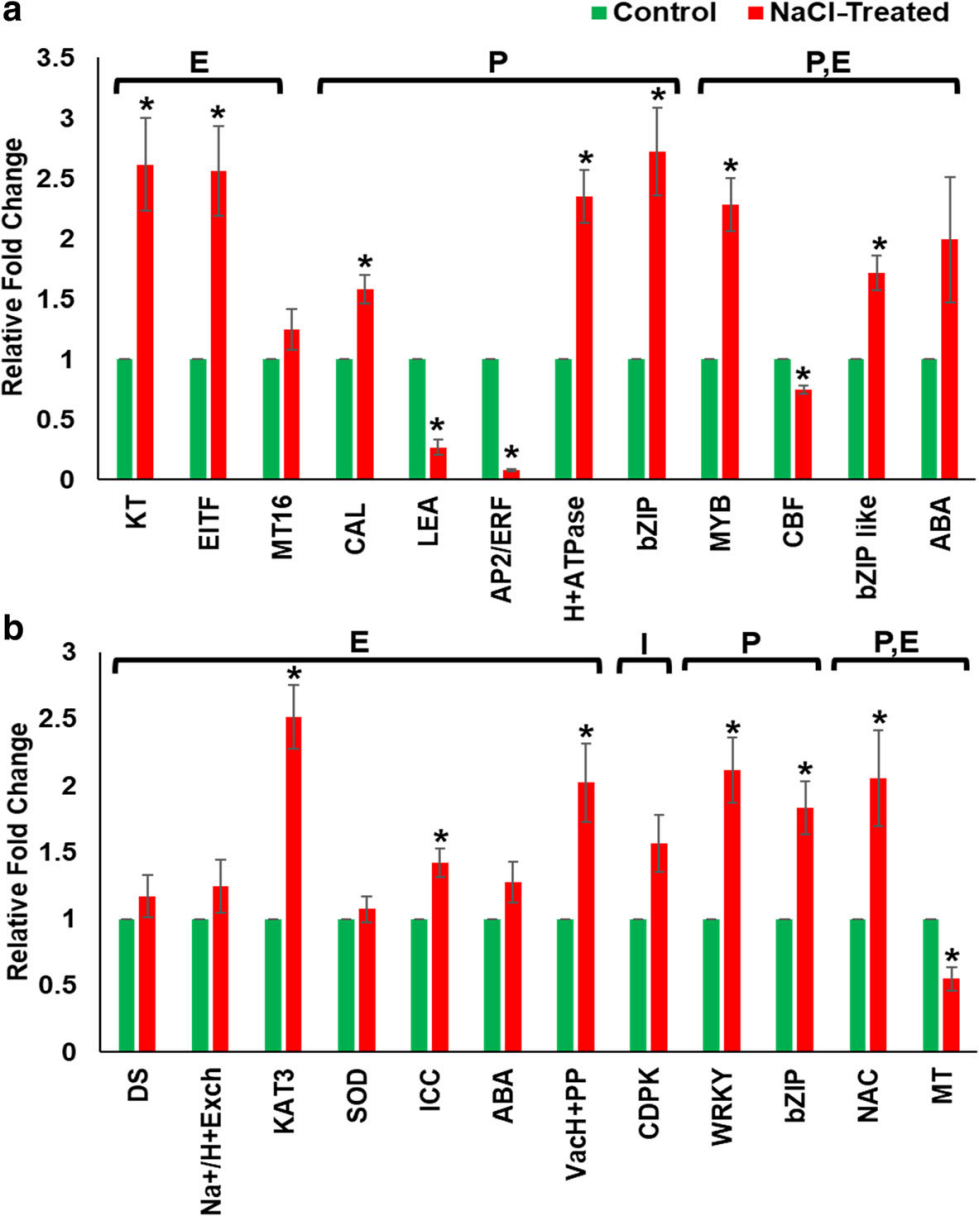
The expression level measured using qPCR of differentially hypermethylated (**a**) and hypomethylated (**b**) genes for the mCG context sequence located within the promoter (P), exon (E) or intron (I) due to salinity stress. Bars represent mean ± SE (*n* = 3). Significant (*p* < 0.05) differences are denoted by asterisk

In order to determine the relationship between mCG methylation at different regions (promoter, exon and intron) and the transcriptome abundance of some salinity-related genes, the expression level of a group of genes was investigated using qPCR. The differentially methylated mCG sites were chosen for this analysis because they have the highest level of methylation among the sequence context sites and, therefore, they may display a clear effect on gene expression. However, the qPCR results did not reveal an unblemished relationship between the level of DNA methylation and gene expression (Fig. 9). Apart from the methylation within the exons, which frequently shows an indeterminate effect on gene expression, promoters with strongly methylated sites usually show a clear effect on gene expression. While the expression level increased in some hypomethylated sites within the promoters, the expression level also increased in other hypermethylated sites of other promoters. As an example, the expression level increased in the hypermethylated basic-region leucine zipper motif (bZIP) transcription factors-like gene (Fig. 9a), and also increased in the hypomethylated bZIP gene (Fig. 9b). However, it was also noted that the expression level of LEA and AP2/ERF genes was significantly (*p* < 0.05) reduced due to the increase in the mCG methylation in their promotor regions, an observation which was consistent with the typical gene expression inhibition feature of methylated promoters in eukaryotes. Therefore, it was difficult to generate a clear idea about the effect of the DNA methylation on gene expression using the available set of data of this study because the possible involvement of other epigenetic agents that can control gene expression under stress conditions.

### Mass spectrometry analysis revealed an increase in global DNA methylation levels in roots exposed to salinity

A pool of DNA samples extracted from the root tissues of saline-treated and control plants was analyzed separately for the relative content of 5mdC and 5HmdC using mass spectrometry. While the 5HmdC levels were undetectable in the DNA samples, analysis showed that DNA extracted from the control roots contained an average amount of 12.9 ± 0.05% (mean ± SD) of 5mdC; however, the same analysis showed that the DNA extracted from the saline-treated roots contained an average amount of 15.4 ± 0.5% (mean ± SD) of 5mdC. Therefore, there was a significant (*p* ≤ 0.05) increase of about 19.4% in 5mdC in the genomic DNA extracted from roots of the plants exposed to salinity.

## Discussion

DNA methylation is part of the stress reaction that takes place in plants in response to suboptimal environmental conditions [47]. We previously showed that salinity stress increases global DNA methylation at the mCG site in *M. truncatula;* however, little information was provided regarding the identity of the methylated genes in response to salinity stress [31]. Using several different methods to detect the methylation dynamics provides a comprehensive impression of these changes in *M. truncatula,* as each methodology generates a different type of knowledge and has its own technical limitations in determining the DNA methylation alterations that occur in the cell due to salinity stress. For example, MSAP can determine the majority of the DNA changes that take place in the double strand CCGG (mCG) site, while WGBS can finely map and quantify the methylation changes at an unbiased genome-wide DNA level in the mCHH, mCHG and mCG sites, and global DNA analysis using mass spectrometry quantifies the lump sum 5mdC and 5HmdC of a particular genome.

The WGBS results obtained from this report are consistent with the fact that the bisulfite conversion reduces the complexity of the genomic sequence and therefore reduces the ability of most computational programs to align sequences onto the reference genome. For instance, while native DNA sequencing for humans typically aligns at 90–95%, bisulfite-converted DNA typically has a mapping efficiency closer to 30–50%, depending on the template [48]. Additionally, the alignment is only as good as the reference genome and the quality of the mapped reads. The *M. truncatula* reference genome has not been revised and, thus, there may still be many gaps or undetermined regions in the genome, which negatively affects the mapping efficiency. mCG coverage is approximately what we would expect for this type of project, at an average of 15X and 18X per site for the control and NaCl-treated samples, respectively. Despite this difference in coverage, there are over 5.7 million mCG sites that were shared between the samples (Table 1).

In the WGBS analysis, DNA was separately extracted from a pool of ten treated and untreated plants. Each pool was considered as a single biological replicate which was used in this analysis. The strategy used in this report is valid and has previously been employed by other WGBS projects focused on plant species [20, 33]. Fortunately, the results obtained from WGBS and the global quantification of 5-mCs obtained using mass spectrometry of *M. truncatula* in response to salinity stress were consistent with those results previously obtained using other DNA methylation detection methods such as MSAP and ELISA, in which several biological and experimental replicates were used [31].

The 5-mC percentage varies based on genotype, tissue, developmental stage and environmental conditions. Unsurprisingly, the 5-mC levels of *M. truncatula* measured by mass spectrometry are within the known ranges of other plant species, which usually fluctuate between 5% and 30% of total cytosines [49, 50]. For example, mass spectrometry analysis of the *Arabidopsis thaliana* leaf samples grown under control environmental conditions revealed that 14.0% of total cytosines were methylated [51].

The mass spectrometry analysis did not reveal a detectable amount of 5HmdC in *M. truncatula*. Indeed, 5HmdC was not previously detected in remarkable amounts in terrestrial plants [27]; however, 5HmdC was detected in significant amounts in other eukaryotic systems. While mammalian cells contained significant amounts of 5HmdC coding for important epigenetic activities [52, 53], the trace amounts of 5HmdC that were previously identified in the Arabidopsis genome could be due to the random oxygen species’ reactivity with 5-mCs, rather than due to a programmed epigenetic regulation [54].

Regardless of plant conditions, the WGBS analysis revealed that the number of the mCHH is highest among the methylated sequence contexts found in the *M. truncatula* genome (Fig. 1, Table 2)*.* Despite the fact that the methylation pattern is conserved among eukaryotes, new studies showed that this conservation is not strict for whole species and tissue types. For example, the relatively abundant proportion of the different methylated sequence contexts in *M. truncatula* was not consistent with the methylome analysis of Arabidopsis, rice or maize [55–57]. However, this relative abundance is consistent with the methylome analysis of *Betula platyphylla* [58].

Global analysis of the chromosomes and the gene body methylation levels of the 5-mC showed that the mCG and mCHG sequence contexts had a higher ratio of methylation than the mCHH, but these methylation levels slightly reduced only at the gene body level in all sequence contexts in response to salinity treatment (Fig. 3). Given the idea that a minor reduction in DNA methylation at promoter regions can increase the gene activity, this reduction in DNA methylation may represent a slight overall increase in gene expression, which might be required by plants to deal with salinity stress. While salinity treatment did not demonstrate an effect on DNA methylation at the chromosome level, the level of mCG and mCHH did demonstrate fluctuation along the chromosomes (Fig. 4), though this is quite normal for the chromosomes. For example, the presence of a high level of DNA methylation in the putative centromere and pericentromere regions of the chromosomes is consistent with other plant species, such as Arabidopsis [14, 59] and black cottonwood [60]. A large number of genes within these regions are likely to be hypermethylated and transcriptionally silent [61, 62].

Despite the fact that the mCHH numbers were the highest among the methylated sequence contexts, the level of methylation for this sequence context was lowest at the chromosomal and gene body levels (Figs. 4 and 5). The reason for the large mCHH numbers could be related to the high probability of occurrence in the genome of the *M. truncatula* due to the redundancy of the asymmetric CHH motif sequence. However, the methylation in that site is generally less stable, perhaps due to the differences in the methylation maintenance mechanisms for different sequence contexts [63, 64].

DNA methylation is often associated with gene expression alterations. However, these alterations are influenced by the location of the methylated region across the gene structures. While hypermethylation at the mCG sites in promoter regions are known as gene silencing marks in eukaryotes, the mCHH sequence contexts are associated with the enrichment of the 24-nt small RNA species (sRNAs) and gene expression [13, 65]. Unlike the exon region, which was enriched with methylated mCG due to salinity stress, the promoter region was enriched with mCHH in *M. truncatula,* based on the analysis of the top 2000 DMSs sites. The hypermethylation of mCHH may have an impact on the regulation of salinity-responsive genes; however, no expression information is available in this particular project to correlate mCHH with the transcription abundance. Indeed, previous studies revealed that methylated mCHH in the maize genome is associated with gene expression [20, 66]. In addition, a fluctuation in the methylation status was observed at the mCHH sites of the promoter sequence of the *ETHYLENE RESPONSIVE FACTOR 6*, *SUPPRESSION OF RVS 161 DELTA 4* and *3-KETOACYL-COA SYNTHASE 13* genes, which control cotton fiber growth [67]. On the other hand, gene expression analysis of methylated genes on mCG sites of the promoters and exons did not show a clear correlation between methylation status and transcriptome abundance (Fig. 9). This could be due to inappropriate sites of methylation within the promoter, which may not affect the affinity of the transcription factor to the binding site. Nevertheless, a recent study showed that methylation of only certain transcription factor binding sites may block transcription factor binding and, therefore, this aspect should not be considered as a general regulatory mechanism of gene expression, at least in human cells [68]. In addition, under stress conditions, it is likely that other epigenetic controllers may involve that may affect the expression level of certain genes [23, 47].

Salinity stress tolerance in plants is controlled by various mechanisms, which are each regulated by a set of genes with a specific function. High percentages of the gene annotation terms of the cDNA included DMSs of the three sequence contexts belonged to energy production, ion binding and membrane protein categories (Additional file 3: Figure S2, Additional file 4: Figure S3, and Additional file 5: Figure S4). This may imply the involvement of these genes in ion binding and transportation procedures, which usually characterize salinity tolerance mechanisms in plants. The enrichment analysis of the top 2000 methylated genes revealed that the majority of the gene ontology terms of mCG DMSs were overrepresented in hypomethylated genes, indicating that salinity stress inhibited mCG methylation in this group of genes, whereas the opposite situation was observed when mCHH DMSs were tested (Fig. 8). However, the gene ontology terms of the mCHG DMSs were enriched with various gene categories, whether the plants grew under control or saline conditions. Therefore, mCHG showed less gene-methylation specificity in response to salinity stress than the other two sequence contexts.

Salinity stress notably increased the mCG methylation of other plant species, such as somatically hybridized wheat [69] and some rice genotypes [70]. However, their contribution to gene activity in salinity tolerance mechanisms is yet to be elucidated. Despite the fact that DNA methylation has some common pattern of distribution and function among the genomes of the plant species, its action may significantly vary among species, and even among the different cultivars of the same species, in response to an environmental stimulus [65].

Purine and thiamine metabolisms and flavonoid biosynthesis were among the pathways enriched with enzymes harboring the DMSs. Given that the expression level may have been influenced by the methylation status, this result is unsurprising since these primary metabolisms have been involved in the salinity tolerance mechanisms of other plant species [71–74]. Nucleotide metabolism in plants is notably affected by environmental stresses, including salinity. However, degraded nucleotide synthesis is recycled via alternative pathways, which requires additional enzymes [75]. Thiamine and flavonoids may act as antioxidant agents in plants grown under salinity conditions [73, 76]. Accordingly, it has been previously shown that the overexpression of thiamine synthesis genes or an application of thiamin on Arabidopsis enhanced salinity stress tolerance [72, 73]. In addition, a number of genes involved in the flavonoid biosynthesis pathway differentially accumulated in rice due to salinity stress [77, 78].

## Conclusions

The results obtained from this report show that salinity promotes global DNA methylation and highlighted, for the first time in *M. truncatula*, the divergent effects of salinity on DNA methylation at base-resolution levels. While the number of the mCG sites was the least and the number of the mCHH sites was the most among the 5-mC identified in the *M. truncatula* genome, the level of the methylation was inversely correlated with the number of each 5-mC sequence context found within the genome. Additionally, the current study showed that the correlations between the expression levels of some potentially important genes in salinity tolerance mechanisms and the changes in the methylation ratio of mCG in response to salinity are imperceptible. Nevertheless, these observations call into question about the actual role of DNA methylation alterations in modulating gene expression under stress conditions however, precise identification of this role requires further intensive investigation, which may eventually aid in the understanding of the complexity of the salinity tolerance mechanisms of this plant.

## Additional files

Additional file 1: Table S1. Oligonucleotides used in the qPCR and the methylation status of the corresponding gene on different component. Promoters were indicated as (P), exon (E) and intron (I). (XLSX 12 kb)

Additional file 2: Figure S1. Clustering heat map analysis of the top 100 methylated sites based on DNA methylation levels of mCG, mCHG and mCHH DMSs. Dendrograms of hierarchical clustering were obtained based on the methylation ratio. Red and yellow color scales represent individual 5-mC sites that are 0% and 100% methylated, respectively. (TIFF 2889 kb)

Additional file 3: Figure S2. Functional annotations of DMSs of the top 2000 altered genes for the mCG sequence context. The annotations and gene ontologies were classified based on the biological process (A), cellular components (B) and molecular functions (C) of the annotated genes. (TIFF 1255 kb)

Additional file 4: Figure S3. Functional annotations of the DMSs of the top 2000 altered genes for the mCHG sequence context. The annotations and gene ontologies were classified based on the biological process (A), cellular components (B) and molecular functions (C) of the annotated genes. (TIFF 1285 kb)

Additional file 5: Figure S4. Functional annotations of the DMSs of the top 2000 altered genes for the mCHH sequence context. The annotations and gene ontologies were classified based on the biological process (A), cellular components (B) and molecular functions (C) of the annotated genes. (TIFF 1264 kb)

Additional file 6: Table S2. Mapping of differentially methylated genes for the mCG sequence context on the metabolic pathways based on annotated coding enzymes. (XLSX 40 kb)

Additional file 7: Table S3. Mapping of differentially methylated genes for the mCHG sequence context onto metabolic pathways based on annotated coding enzymes. (XLSX 40 kb)

Additional file 8: Table S4. Mapping of differentially methylated genes for the mCHH sequence context onto metabolic pathways based on annotated coding enzymes. (XLSX 35 kb)

Additional file 9: Figure S5. Classification of the enzymes coded by gene-harbored DMSs for the mCG (A), mCHG (B) and mCHH (C) contexts. (TIFF 380 kb)

Additional file 10: Table S5. Functional annotation and gene ontology of differentially methylated genes for the mCG sequence context based on coding sequences. (XLSX 425 kb)

Additional file 11: Table S6. Functional annotation and gene ontology of differentially methylated genes for the mCHG sequence context based on coding sequences. (XLSX 463 kb)

Additional file 12: Table S7. Functional annotation and gene ontology of differentially methylated genes for the mCHH sequence context based on coding sequences. (XLSX 417 kb)

## Abbreviations

5HmdC: 5-hydroxymethyl-2′-deoxycytidine
5-mCs: 5-methylcytosine nucleotide
5mdC: 5-methyl-2′-deoxycytidine
DEGs: Differentially Expressed Genes
dG: 2′-deoxyguanosine
DMRs: Differentially methylated regions
DMSs: Differentially methylated sites
dS.m^− 1^: Deci Siemens per meter
FRD: False discovery rate
GO: Gene ontology
KEGG: Kyoto Encyclopedia of Genes and Genomes
MSAP: Methylation-sensitive amplified polymorphism
qPCR: Reverse transcriptase-quantitative polymerase chain reaction

## References

[1] Yaish MW, Patankar HV, Assaha DV, Zheng Y, Al-Yahyai R, Sunkar R (2017). Genome-wide expression profiling in leaves and roots of date palm (*Phoenix dactylifera* L.) exposed to salinity. BMC Genomics.

[2] Assaha DV, Ueda A, Saneoka H, Al-Yahyai R, Yaish MW (2017). The role of Na+ and K+ transporters in salt stress adaptation in Glycophytes. Front Physiol.

[3] Al Kharusi L, Assaha DV, Al-Yahyai R, Yaish MW (2017). Screening of date palm (*Phoenix dactylifera* L.) cultivars for salinity tolerance. Forests.

[4] Holliday R, Pugh J (1975). DNA modification mechanisms and gene activity during development. Science.

[5] Riggs AD (1975). X inactivation, differentiation, and DNA methylation. Cytogenet Genome Res.

[6] Waterhouse PM, Wang M-B, Lough T (2001). Gene silencing as an adaptive defence against viruses. Nature.

[7] Ikeuchi M, Iwase A, Sugimoto K (2015). Control of plant cell differentiation by histone modification and DNA methylation. Curr Opin Plant Biol.

[8] Jones PA, Takai D (2001). The role of DNA methylation in mammalian epigenetics. Science.

[9] Beard C, Li E, Jaenisch R (1995). Loss of methylation activates Xist in somatic but not in embryonic cells. Genes Dev.

[10] Fedoroff NV (2012). Transposable elements, epigenetics, and genome evolution. Science.

[11] Demeulemeester M, Van Stallen N, De Proft M (1999). Degree of DNA methylation in chicory (*Cichorium intybus* L.): influence of plant age and vernalization. Plant Sci.

[12] Yaish M (2017). Epigenetic modifications associated with Abiotic and biotic stresses in plants: an implication for understanding plant evolution. Front Plant Sci.

[13] Stroud H, Do T, Du J, Zhong X, Feng S, Johnson L (2014). Non-CG methylation patterns shape the epigenetic landscape in Arabidopsis. Nat Struct Mol Biol.

[14] Law JA, Jacobsen SE (2010). Establishing, maintaining and modifying DNA methylation patterns in plants and animals. Nat Rev Genet.

[15] Chan SW, Henderson IR, Jacobsen SE (2005). Gardening the genome: DNA methylation in Arabidopsis thaliana. Nat Rev Genet.

[16] Wang J, Marowsky NC, Fan C (2014). Divergence of gene body DNA methylation and evolution of plant duplicate genes. PLoS One.

[17] Bell AC, Felsenfeld G (2000). Methylation of a CTCF-dependent boundary controls imprinted expression of the Igf2 gene. Nature.

[18] Zhang X, Yazaki J, Sundaresan A, Cokus S, Chan SW-L, Chen H (2006). Genome-wide high-resolution mapping and functional analysis of DNA methylation in Arabidopsis. Cell.

[19] Li X, Zhu J, Hu F, Ge S, Ye M, Xiang H (2012). Single-base resolution maps of cultivated and wild rice methylomes and regulatory roles of DNA methylation in plant gene expression. BMC Genomics.

[20] Lu X, Wang W, Ren W, Chai Z, Guo W, Chen R (2015). Genome-wide epigenetic regulation of gene transcription in maize seeds. PLoS One.

[21] Bilichak A, Kovalchuk I (2016). Transgenerational response to stress in plants and its application for breeding. J Exp Bot.

[22] Yaish MW, Sunkar R, Zheng Y, Ji B, Al-Yahyai R, Farooq SA (2015). A genome-wide identification of the miRNAome in response to salinity stress in date palm (*Phoenix dactylifera* L.). Front Plant Sci.

[23] Yaish MW. DNA methylation-associated epigenetic changes in stress tolerance of plants. In: Molecular stress physiology of plants*.* Springer; New Delhi. 2013: 427-440. doi.org/10.1007/978-81-322-0807-5_17.

[24] Ferreira LJ, Azevedo V, Maroco J, Oliveira MM, Santos AP (2015). Salt tolerant and sensitive rice varieties display differential methylome flexibility under salt stress. PLoS One.

[25] Lewsey MG, Hardcastle TJ, Melnyk CW, Molnar A, Valli A, Urich MA (2016). Mobile small RNAs regulate genome-wide DNA methylation. Proc Natl Acad Sci U S A.

[26] Feng S, Jacobsen SE, Reik W (2010). Epigenetic reprogramming in plant and animal development. Science.

[27] Erdmann RM, Souza AL, Clish CB, Gehring M (2015). 5-Hydroxymethylcytosine is not present in appreciable quantities in Arabidopsis DNA. G3.

[28] Lei M, Zhang H, Julian R, Tang K, Xie S, Zhu J-K (2015). Regulatory link between DNA methylation and active demethylation in Arabidopsis. Proc Natl Acad Sci U S A.

[29] Satgé C, Moreau S, Sallet E, Lefort G, Auriac M-C, Remblière C (2016). Reprogramming of DNA methylation is critical for nodule development in *Medicago truncatula*. Nature Plants.

[30] Barker DG, Bianchi S, Blondon F, Dattée Y, Duc G, Essad S (1990). *Medicago truncatula*, a model plant for studying the molecular genetics of the Rhizobium-legume symbiosis. Plant Mol Biol Report.

[31] Al-Lawati A, Al-Bahry S, Victor R, Al-Lawati AH, Yaish MW. Salt stress alters DNA methylation levels in alfalfa (*Medicago* spp). Genet Mol Res. 2016;15(1) 10.4238/gmr.15018299. 10.4238/gmr.15018299 26985924

[32] Yaish MW, Peng M, Rothstein SJ (2014). Global DNA methylation analysis using methyl-sensitive amplification polymorphism (MSAP). Methods Mol Biol.

[33] Zhong S, Fei Z, Chen YR, Zheng Y, Huang M, Vrebalov J (2013). Single-base resolution methylomes of tomato fruit development reveal epigenome modifications associated with ripening. Nat Biotechnol.

[34] Al-Harrasi I, Al-Yahyai R, Yaish MW. Detection of differential DNA Methylation DNA methylation under stress conditions using Bisulfite sequence analysis. In: Sunkar R. (eds) Plant Stress Tolerance. Methods molecular biology, vol. 1631. New York: Humana Press; 2017. 10.1007/978-1-4939-7136-7_7. 10.1007/978-1-4939-7136-7_7 28735394

[35] Krueger F, Andrews SR (2011). Bismark: a flexible aligner and methylation caller for Bisulfite-Seq applications. Bioinformatics.

[36] Ware D, Jaiswal P, Ni J, Pan X, Chang K, Clark K (2002). Gramene: a resource for comparative grass genomics. Nucleic Acids Res.

[37] Gotz S, Garcia-Gomez JM, Terol J, Williams TD, Nagaraj SH, Nueda MJ (2008). High-throughput functional annotation and data mining with the Blast2GO suite. Nucleic Acids Res.

[38] Kanehisa M, Goto S, Kawashima S, Okuno Y, Hattori M (2004). The KEGG resource for deciphering the genome. Nucleic Acids Res.

[39] Krzywinski M, Schein J, Birol I, Connors J, Gascoyne R, Horsman D (2009). Circos: an information aesthetic for comparative genomics. Genome Res.

[40] Song L, James SR, Kazim L, Karpf AR (2005). Specific method for the determination of genomic DNA methylation by liquid chromatography-electrospray ionization tandem mass spectrometry. Anal Chem.

[41] Bustin SA, Benes V, Garson JA, Hellemans J, Huggett J, Kubista M (2009). The MIQE guidelines: minimum information for publication of quantitative real-time PCR experiments. Clin Chem.

[42] Livak KJ, Schmittgen TD (2001). Analysis of relative gene expression data using real-time quantitative PCR and the 2^− ΔΔCT^ method. Methods.

[43] Kuppusamy KT, Endre G, Prabhu R, Penmetsa RV, Veereshlingam H, Cook DR (2004). LIN, a *Medicago truncatula* gene required for nodule differentiation and persistence of rhizobial infections. Plant Physiol.

[44] Li Y, Chen X, Chen Z, Cai R, Zhang H, Xiang Y (2016). Identification and expression analysis of BURP domain-containing genes in *Medicago truncatula*. Front Plant Sci.

[45] Clèries R, Galvez J, Espino M, Ribes J, Nunes V, de Heredia ML (2012). BootstRatio: a web-based statistical analysis of fold-change in qPCR and RT-qPCR data using resampling methods. Comput Biol Med.

[46] Yaish MW (2015). Proline accumulation is a general response to abiotic stress in the date palm tree (*Phoenix dactylifera* L.). Genet Mol Res.

[47] Yaish MW, Colasanti J, Rothstein SJ (2011). The role of epigenetic processes in controlling flowering time in plants exposed to stress. J Exp Bot.

[48] Tran H, Porter J, Sun M-a, Xie H, Zhang L (2014). Objective and comprehensive evaluation of bisulfite short read mapping tools. Adv Bioinforma.

[49] Wagner I, Capesius I (1981). Determination of 5-methylcytosine from plant DNA by high-performance liquid chromatography. Biochim Biophys Acta, Nucleic Acids Protein Synth.

[50] Leutwiler LS, Hough-Evans BR, Meyerowitz EM (1984). The DNA of *Arabidopsis thaliana*. Mol Gen Genet.

[51] Capuano F, Mülleder M, Kok R, Blom HJ, Ralser M (2014). Cytosine DNA methylation is found in *Drosophila melanogaster* but absent in *Saccharomyces cerevisiae*, *Schizosaccharomyces pombe*, and other yeast species. Anal Chem.

[52] Ko M, Huang Y, Jankowska AM, Pape UJ, Tahiliani M, Bandukwala HS (2010). Impaired hydroxylation of 5-methylcytosine in myeloid cancers with mutant TET2. Nature.

[53] Jin S-G, Jiang Y, Qiu R, Rauch TA, Wang Y, Schackert G (2011). 5-Hydroxymethylcytosine is strongly depleted in human cancers but its levels do not correlate with IDH1 mutations. Cancer Res.

[54] Liu S, Dunwell TL, Pfeifer GP, Dunwell JM, Ullah I, Wang Y (2013). Detection of oxidation products of 5-methyl-2′-deoxycytidine in Arabidopsis DNA. PLoS One.

[55] Zemach A, McDaniel IE, Silva P, Zilberman D (2010). Genome-wide evolutionary analysis of eukaryotic DNA methylation. Science.

[56] Feng S, Cokus SJ, Zhang X, Chen P-Y, Bostick M, Goll MG (2010). Conservation and divergence of methylation patterning in plants and animals. Proc Natl Acad Sci U S A.

[57] Li Q, Song J, West PT, Zynda G, Eichten SR, Vaughn MW (2015). Examining the causes and consequences of context-specific differential DNA methylation in maize. Plant Physiol.

[58] Su C, Wang C, He L, Yang C, Wang Y (2014). Shotgun Bisulfite sequencing of the Betula Platyphylla genome reveals the Tree's DNA Methylation patterning. Int J Mol Sci.

[59] Gehring M, Bubb KL, Henikoff S (2009). Extensive demethylation of repetitive elements during seed development underlies gene imprinting. Science.

[60] Vining KJ, Pomraning KR, Wilhelm LJ, Priest HD, Pellegrini M, Mockler TC (2012). Dynamic DNA cytosine methylation in the *Populus trichocarpa* genome: tissue-level variation and relationship to gene expression. BMC Genomics.

[61] Zilberman D, Gehring M, Tran RK, Ballinger T, Henikoff S (2007). Genome-wide analysis of *Arabidopsis thaliana* DNA methylation uncovers an interdependence between methylation and transcription. Nature Genet.

[62] May BP, Lippman ZB, Fang Y, Spector DL, Martienssen RA (2005). Differential regulation of strand-specific transcripts from Arabidopsis centromeric satellite repeats. PLoS Genet.

[63] Dalakouras A, Dadami E, Zwiebel M, Krczal G, Wassenegger M (2012). Transgenerational maintenance of transgene body CG but not CHG and CHH methylation. Epigenetics.

[64] Weinhold A, Kallenbach M, Baldwin IT (2013). Progressive 35S promoter methylation increases rapidly during vegetative development in transgenic *Nicotiana attenuata* plants. BMC Plant Biol.

[65] Garg R, Chevala VN, Shankar R, Jain M (2015). Divergent DNA methylation patterns associated with gene expression in rice cultivars with contrasting drought and salinity stress response. Sci Rep.

[66] Gent JI, Ellis NA, Guo L, Harkess AE, Yao Y, Zhang X (2013). CHH islands: de novo DNA methylation in near-gene chromatin regulation in maize. Genome Res.

[67] Jin X, Pang Y, Jia F, Xiao G, Li Q, Zhu Y (2013). A potential role for CHH DNA methylation in cotton fiber growth patterns. PLoS One.

[68] Medvedeva YA, Khamis AM, Kulakovskiy IV, Ba-Alawi W, Bhuyan MSI, Kawaji H, et al. Effects of cytosine methylation on transcription factor binding sites. BMC Genomics. 2014;15(1) 10.1186/1471-2164-15-119. 10.1186/1471-2164-15-119 PMC3986887 24669864

[69] Wang M, Qin L, Xie C, Li W, Yuan J, Kong L (2014). Induced and constitutive DNA methylation in a salinity tolerant wheat introgression line. Plant Cell Physiol.

[70] Karan R, DeLeon T, Biradar H, Subudhi PK (2012). Salt stress induced variation in DNA methylation pattern and its influence on gene expression in contrasting rice genotypes. PLoS One.

[71] Nam MH, Huh SM, Kim KM, Park WW, Seo JB, Cho K (2012). Comparative proteomic analysis of early salt stress-responsive proteins in roots of SnRK2 transgenic rice. Proteome Sci.

[72] Rapala-Kozik M, Wolak N, Kujda M, Banas AK (2012). The upregulation of thiamine (vitamin B 1) biosynthesis in *Arabidopsis thaliana* seedlings under salt and osmotic stress conditions is mediated by abscisic acid at the early stages of this stress response. BMC Plant Biol.

[73] Tunc-Ozdemir M, Miller G, Song L, Kim J, Sodek A, Koussevitzky S (2009). Thiamin confers enhanced tolerance to oxidative stress in Arabidopsis. Plant Physiol.

[74] Xie Y, Mao Y, Duan X, Zhou H, Lai D, Zhang Y (2015). Arabidopsis HY1-modulated Stomatal movement: an integrative hub for its functionally associated with ABI4 in the dehydration-induced ABA responsiveness. Plant Physiol.

[75] Witz S, Jung B, Fürst S, Möhlmann T (2012). *De novo* pyrimidine nucleotide synthesis mainly occurs outside of plastids, but a previously undiscovered nucleobase importer provides substrates for the essential salvage pathway in Arabidopsis. Plant Cell.

[76] Agati G, Azzarello E, Pollastri S, Tattini M (2012). Flavonoids as antioxidants in plants: location and functional significance. Plant Sci.

[77] Walia H, Wilson C, Condamine P, Liu X, Ismail AM, Zeng L (2005). Comparative transcriptional profiling of two contrasting rice genotypes under salinity stress during the vegetative growth stage. Plant Physiol.

[78] Ithal N, Reddy AR (2004). Rice flavonoid pathway genes, *OsDfr* and *OsAns,* are induced by dehydration, high salt and ABA, and contain stress responsive promoter elements that interact with the transcription activator, OsC1-MYB. Plant Sci.

