# Supplementary figures and images for: Genome-wide DNA Methylation analysis in response to salinity in the model plant caliph medic (Medicago truncatula)
Source: BMC Genomics. 2018 Jan 24;19:78. doi: 10.1186/s12864-018-4484-5 (PMC5781308; doi:10.1186/s12864-018-4484-5)

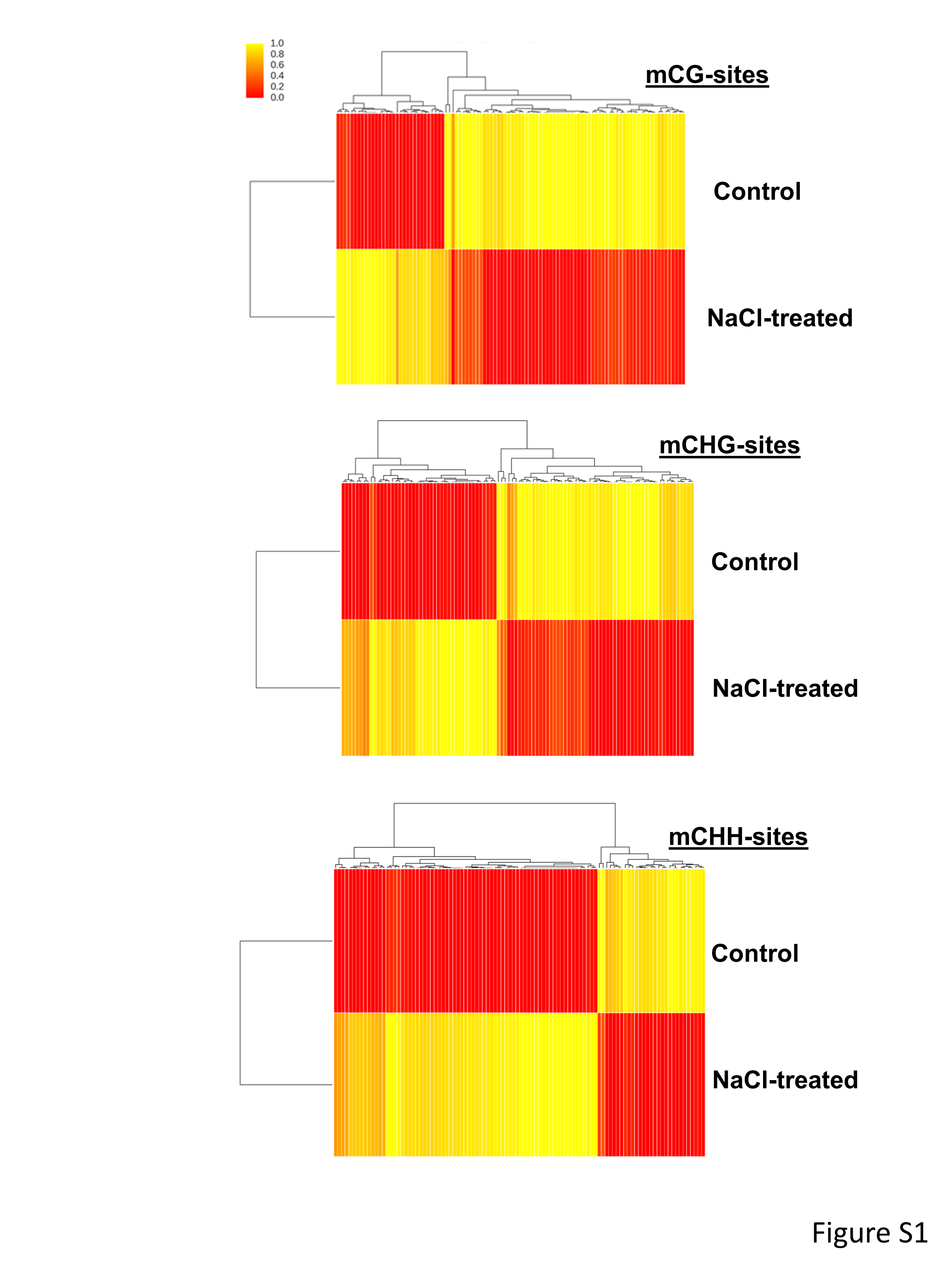

Supplement: Supplementary file 2 — Clustering heat map analysis of the top 100 methylated sites based on DNA methylation levels of mCG, mCHG and mCHH DMSs. Dendrograms of hierarchical clustering were obtained based on the methylation ratio. Red and yellow color scales represent individual 5-mC sites that are 0% and 100% methylated, respectively. (TIFF 2889 kb) [file 12864_2018_4484_MOESM2_ESM.tif]

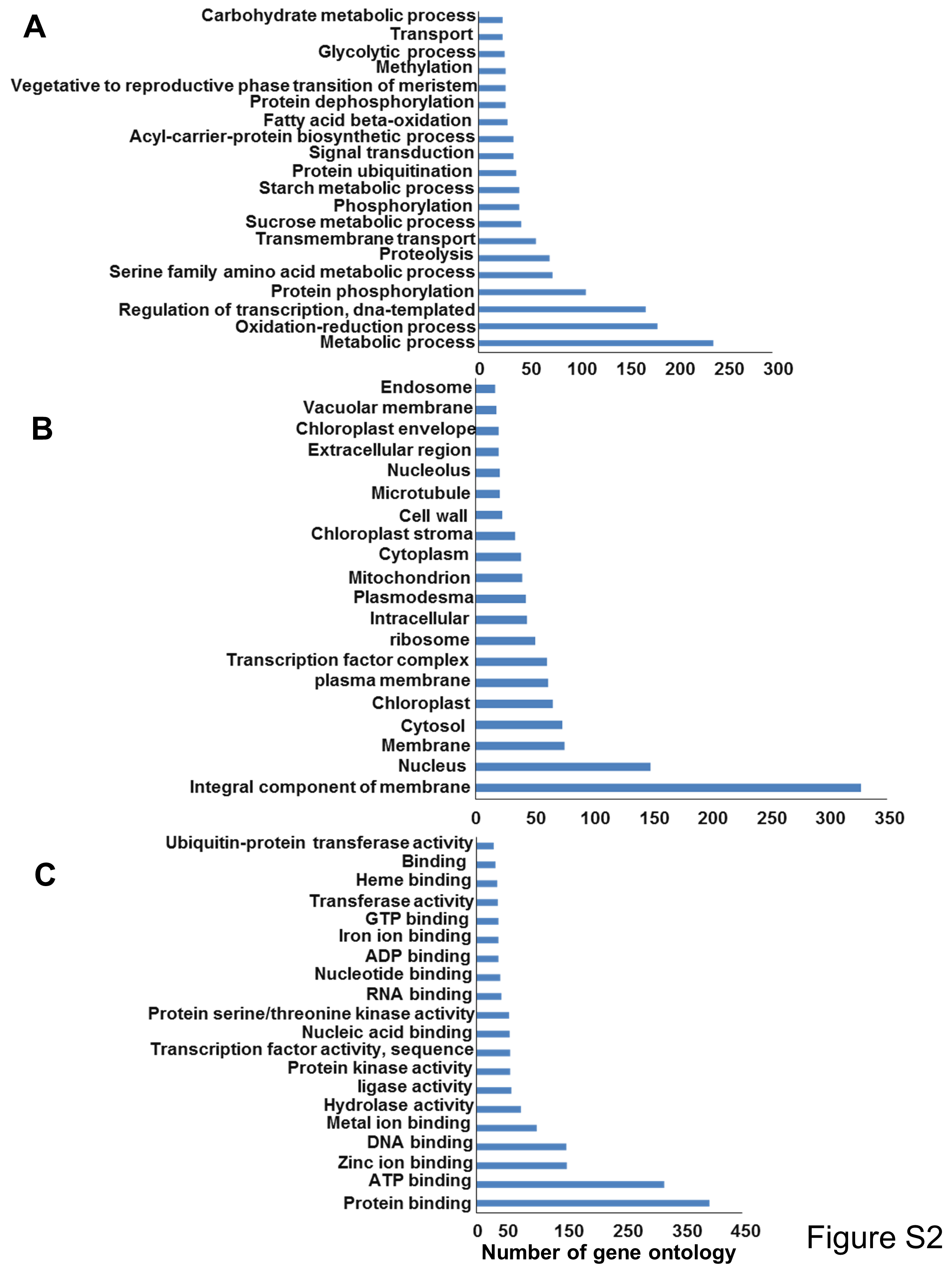

Supplement: Supplementary file 3 — Functional annotations of DMSs of the top 2000 altered genes for the mCG sequence context. The annotations and gene ontologies were classified based on the biological process (A), cellular components (B) and molecular functions (C) of the annotated genes. (TIFF 1255 kb) [file 12864_2018_4484_MOESM3_ESM.tif]

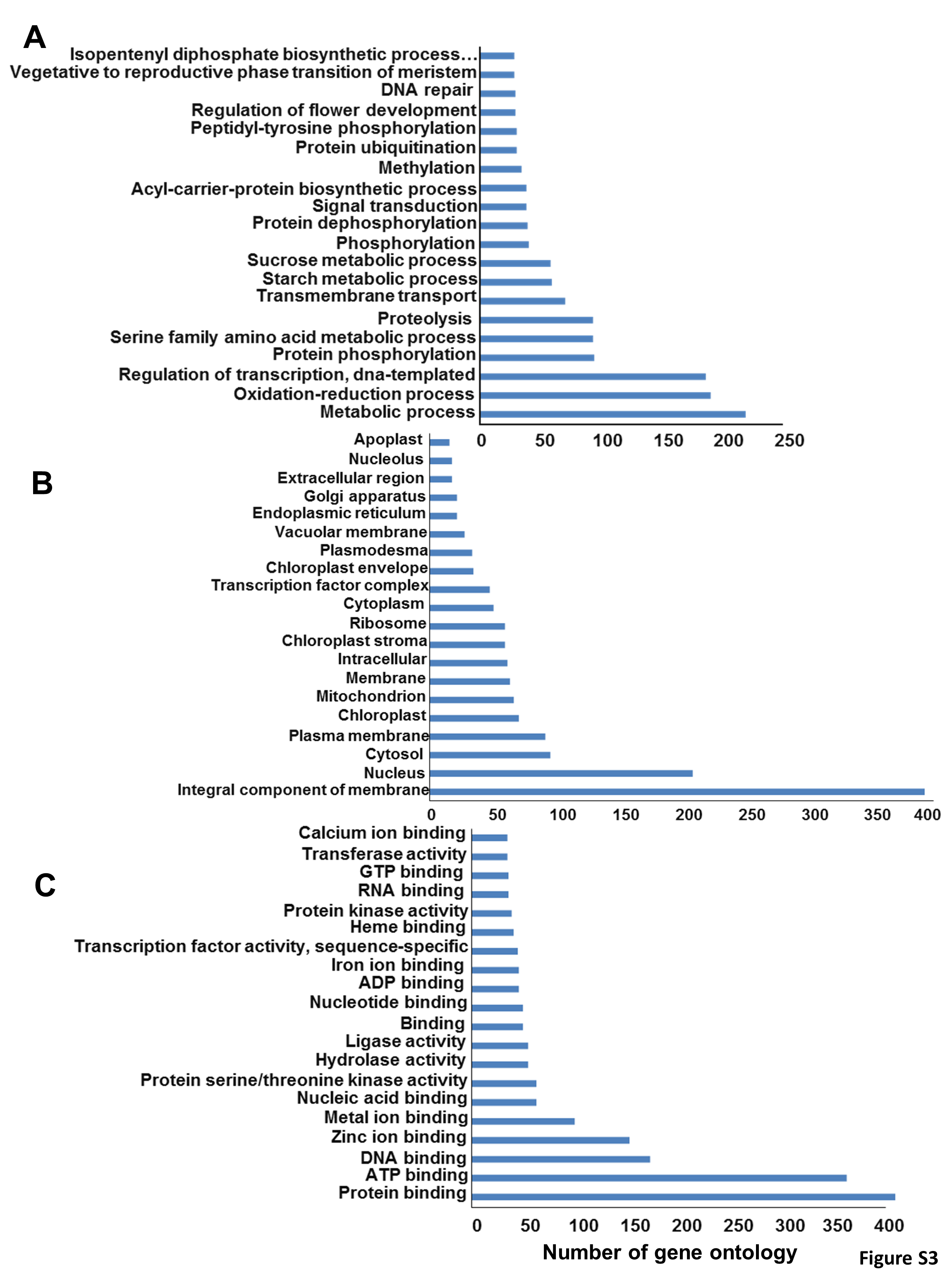

Supplement: Supplementary file 4 — Functional annotations of the DMSs of the top 2000 altered genes for the mCHG sequence context. The annotations and gene ontologies were classified based on the biological process (A), cellular components (B) and molecular functions (C) of the annotated genes. (TIFF 1285 kb) [file 12864_2018_4484_MOESM4_ESM.tif]

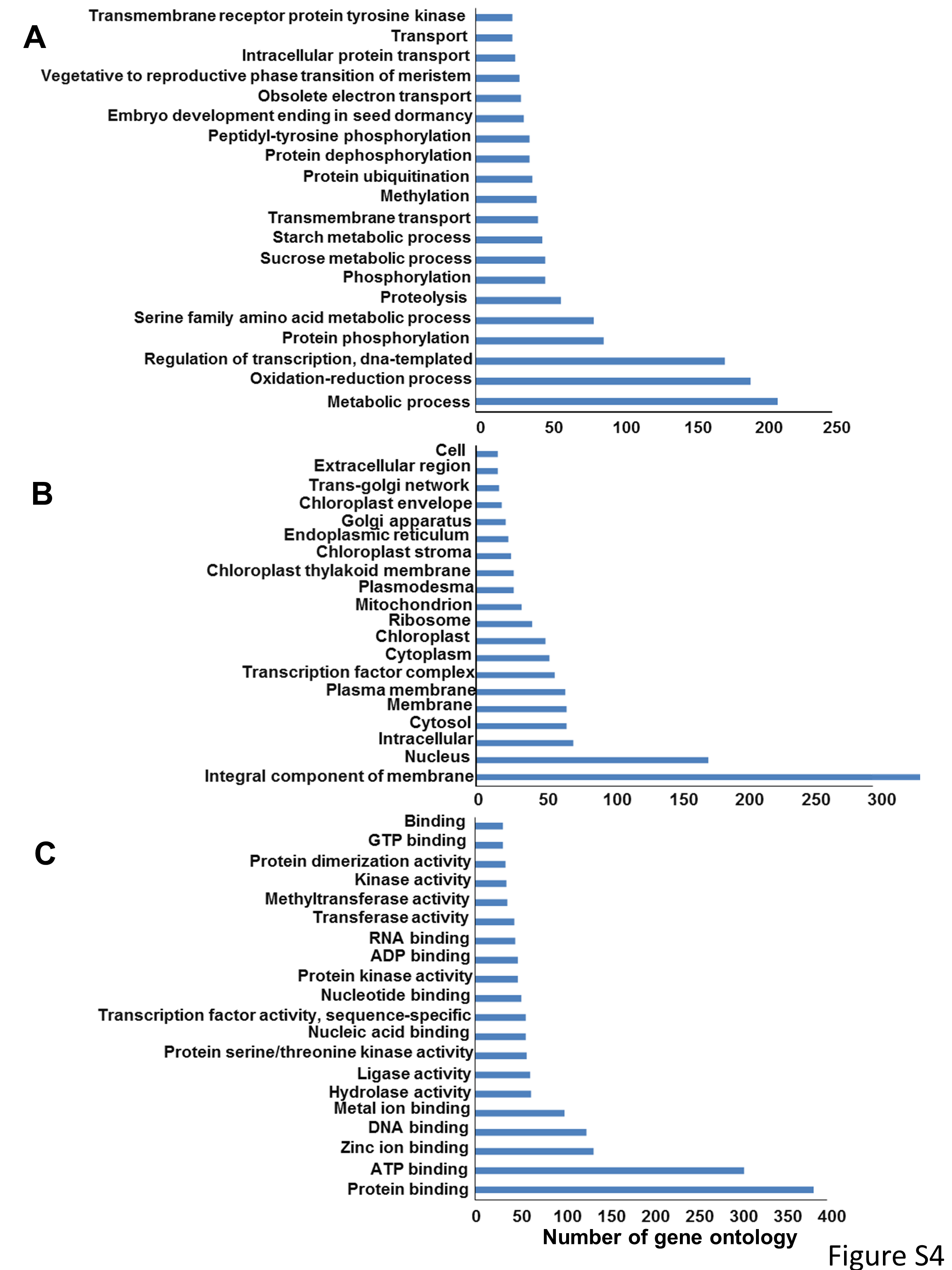

Supplement: Supplementary file 5 — Functional annotations of the DMSs of the top 2000 altered genes for the mCHH sequence context. The annotations and gene ontologies were classified based on the biological process (A), cellular components (B) and molecular functions (C) of the annotated genes. (TIFF 1264 kb) [file 12864_2018_4484_MOESM5_ESM.tif]

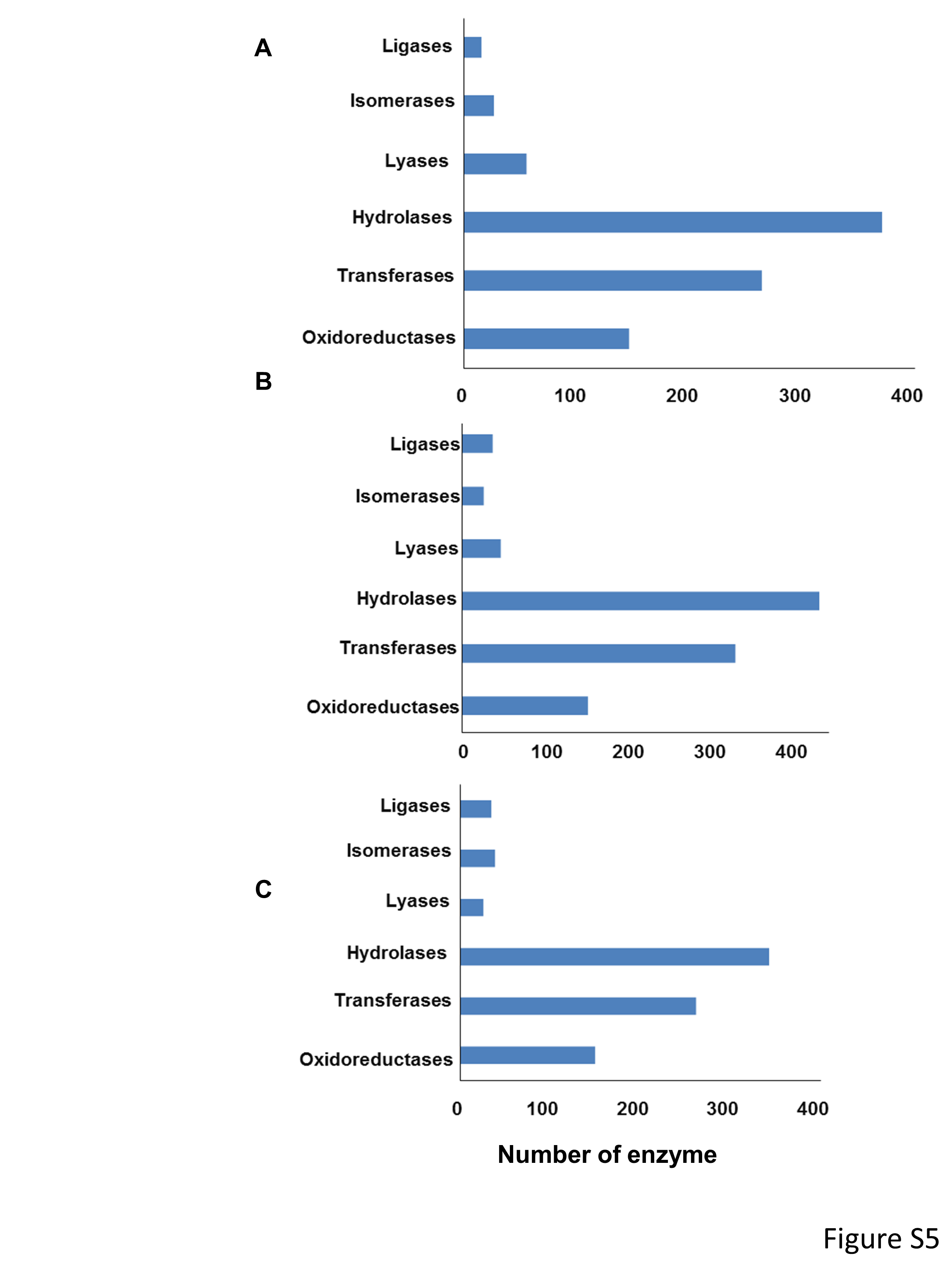

Supplement: Supplementary file 9 — Classification of the enzymes coded by gene-harbored DMSs for the mCG (A), mCHG (B) and mCHH (C) contexts. (TIFF 380 kb) [file 12864_2018_4484_MOESM9_ESM.tif]
